# Unraveling the underlying pathogenic factors driving nonalcoholic steatohepatitis and hepatocellular carcinoma: an in-depth analysis of prognostically relevant gene signatures in hepatocellular carcinoma

**DOI:** 10.1186/s12967-024-04885-6

**Published:** 2024-01-18

**Authors:** Yuan Ni, Maoqing Lu, Ming Li, Xixi Hu, Feng Li, Yan Wang, Dong Xue

**Affiliations:** 1 College of Integrated Chinese and Western Medicine (College of Life Sciences), Anhui University of Chinese Medicine, Hefei, China; 2 State Key Laboratory of Pharmaceutical Biotechnology, School of Life Sciences, Nanjing University, Nanjing, China

**Keywords:** Nonalcoholic steatohepatitis (NASH), Hepatocellular carcinoma (HCC), Macrophages, Scissor algorithm, Clinical predictive mode

## Abstract

**Background:**

Nonalcoholic steatohepatitis (NASH) is a progressive manifestation of nonalcoholic fatty liver disease (NAFLD) that can lead to fibrosis, cirrhosis, and hepatocellular carcinoma (HCC). Despite the growing knowledge of NASH and HCC, the association between the two conditions remains to be fully explored. Bioinformatics has emerged as a valuable approach for identifying disease-specific feature genes, enabling advancements in disease prediction, prevention, and personalized treatment strategies.

**Materials and methods:**

In this study, we utilized CellChat, copy number karyotyping of aneuploid tumors (CopyKAT), consensus Non-negative Matrix factorization (cNMF), Gene set enrichment analysis (GSEA), Gene set variation analysis (GSVA), Monocle, spatial co-localization, single sample gene set enrichment analysis (ssGSEA), Slingshot, and the Scissor algorithm to analyze the cellular and immune landscape of NASH and HCC. Through the Scissor algorithm, we identified three cell types correlating with disease phenotypic features and subsequently developed a novel clinical prediction model using univariate, LASSO, and multifactor Cox regression.

**Results:**

Our results revealed that macrophages are a significant pathological factor in the development of NASH and HCC and that the macrophage migration inhibitory factor (MIF) signaling pathway plays a crucial role in cellular crosstalk at the molecular level. We deduced three prognostic genes (YBX1, MED8, and KPNA2), demonstrating a strong diagnostic capability in both NASH and HCC.

**Conclusion:**

These findings shed light on the pathological mechanisms shared between NASH and HCC, providing valuable insights for the development of novel clinical strategies.

**Supplementary Information:**

The online version contains supplementary material available at 10.1186/s12967-024-04885-6.

## Introduction

Nonalcoholic fatty liver disease (NAFLD) is the primary cause of chronic liver disease, and its prevalence is rapidly increasing worldwide [1]. The spectrum of NAFLD pathology ranges from benign hepatosteatosis to nonalcoholic steatohepatitis (NASH), characterized by significant steatosis, lobular inflammation, programmed cell death, and fibrosis, all of which increase the risk of progression to liver cirrhosis and hepatocellular carcinoma (HCC) [2, 3]. HCC is often associated with the advanced stage of NASH [4, 5]. The prevalence of NASH-HCC is on the rise in Western nations, and HCC has now become the fourth leading cause of cancer-related death [6, 7].

Several studies have suggested a strong association between NASH and HCC [8–11]. Boslem and Zhang have demonstrated that liver inflammation and long-term fibrosis might result in the development of HCC [12, 13]. Recent studies on HCC caused by NASH have shown that endoplasmic reticulum (ER) stress [12], along with metabolic and immune dysfunction, are important factors in its advancement [14]. Research on NASH and HCC has advanced to the genetic molecular level [15]. Previous studies have been made in identifying the genetic components that contribute to NASH and HCC. Nevertheless, early detection of HCC may not occur in a timely manner to allow for successful clinical intervention [16]. The present diagnostic methods for NASH and HCC remain limited. Although invasive liver biopsy has some drawbacks, it is still widely recognized as the most dependable method for detection and is considered the benchmark [17, 18]. During the past decade, the rapid advancement of bioinformatics technology has provided unprecedented opportunities for in-depth analysis and understanding of several diseases, including NASH and HCC [19].

In recent years, there has been growing interest in studying NASH and HCC at the single-cell level [20]. Obtaining a thorough grasp of the cellular-level immunological features of NASH and HCC could lead to enhanced clinical comprehension. This study employed a fusion of single-cell data obtained from individuals with NASH and HCC, as well as bulk RNA-seq data, to examine the immunological attributes of both NASH and HCC. The findings demonstrated that the macrophage migration inhibitory factor (MIF) signaling pathway had a substantial impact on intercellular communication at the single-cell level. Furthermore, macrophages played a vital role in the progression of NASH and HCC. The Scissors algorithm was employed to develop a clinical prediction model that detected positive cell types expressing three separate prognostic genes. The model demonstrated positive diagnostic results in both the NASH and HCC groups. In summary, our research provides new insights into the relationship between NASH and HCC and supports the development of an independent predictive model for these conditions.

## Materials and methods

### Data source

#### Mouse model data sources

We obtained bulk transcriptome data (GSE199105, including only samples from the CHOW and HFD groups) [21] and single-cell transcriptome data (GSE129516) [22] for NASH from the Gene Expression Omnibus (GEO) database (https://www.ncbi.nlm.nih.gov/geo/). We acquired bulk transcriptome data (GSE50431, only the normal hepatocyte and HCC mice were selected) and single-cell transcriptome data (GSE142868, only control Miz1F/F mice were selected) [23] for HCC from the GEO database.

#### Human data sources

**Fig. 1:**
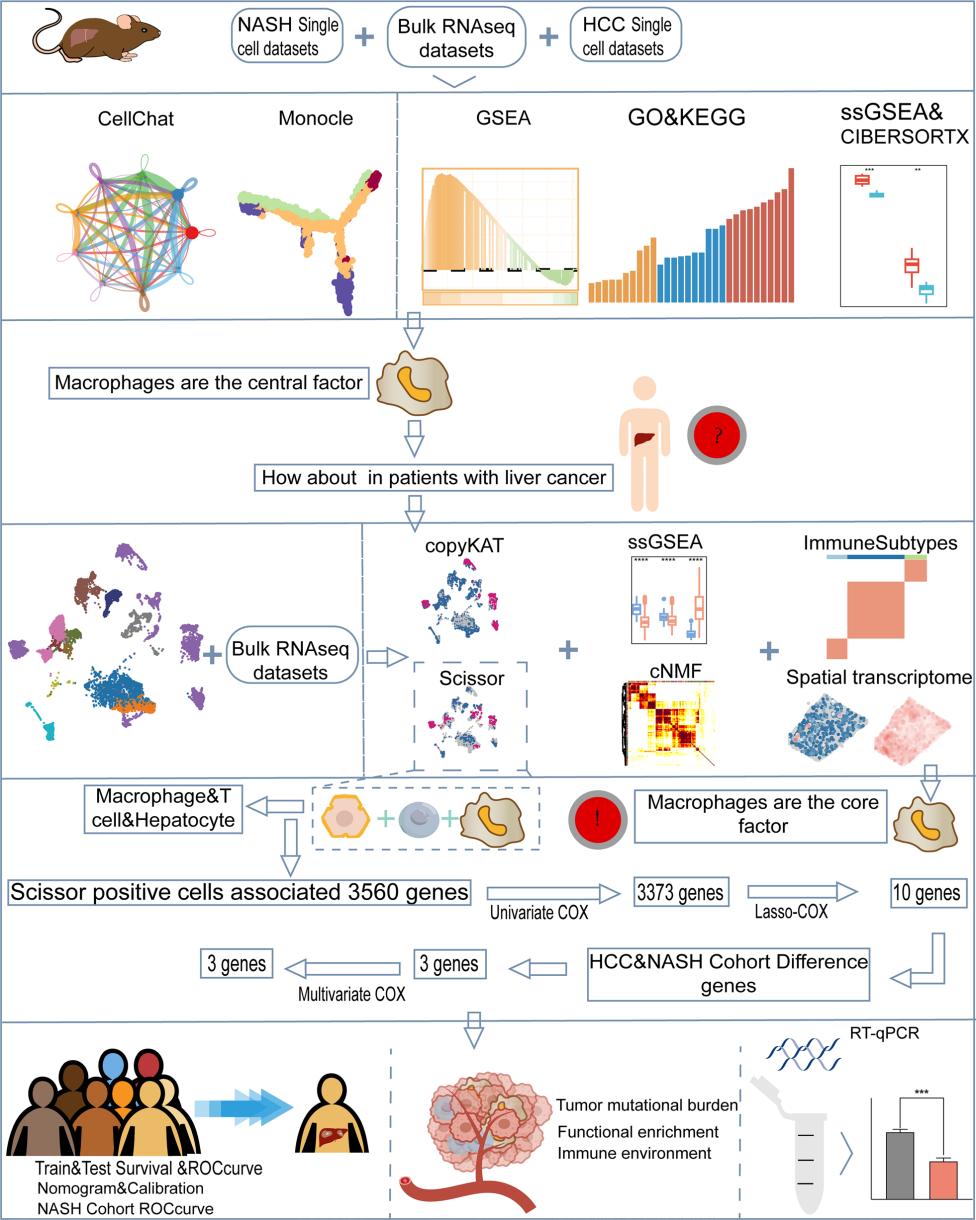
Flowchart of this study

The datasets containing human single-cell transcriptome data can be accessed in GSE151530 [24]. Furthermore, we utilized expression data from The Cancer Genome Atlas (TCGA-LIHC) (https://portal.gdc.cancer.gov/) for HCC patients as the training dataset, and expression data from the International Cancer Genome Consortium (ICGC-LIRI-JP) (https://dcc.icgc.org/) as the validation dataset. Furthermore, four spatial transcriptomic datasets are available in the Zenodo database (https://zenodo.org/) [25]. The study cohort for NASH was derived from the GSE89632 dataset [26]. Figure 1 displays the flow-process diagram that demonstrates the methods employed in our investigation.

### Sample extraction

The gene expression data obtained from the GEO, TCGA, and ICGC databases, originally in FPKM format, were transformed into TPM data. The TPM data underwent a logarithmic transformation and was standardized using the normalizeBetweenArrays function from the limma R package (version 3.54.2) [27]. The gene expression data acquired from GSE50431 was displayed in a format that was not FPKM and had been standardized beforehand. Consequently, the data was subjected to a log2 transformation and subsequently utilized in the subsequent investigations following normalization using the normalizeBetweenArrays function.

Analyzed using the Seurat R package (version 4.3.0) [28], the single-cell transcriptome data from NASH mice (GSE129516), HCC mice (GSE142868), and HCC patients (GSE151530) were examined. The analysis pipeline consisted of several consecutive stages. First, a data filtering process was carried out, where genes were required to show expression in a minimum of three cells, and cells were required to express a varying number of genes ranging from 200 to 10,000. In addition, cells with a mitochondrial gene expression fraction over 20% were eliminated. Subsequently, the LogNormalize and ScaleData functions were utilized for normalization. The FindVariableFeatures tool was employed to detect genes exhibiting significant variability. The FindNeighbors and FindClusters routines were used to perform the ensuing clustering analysis, utilizing these genes. The study explored different clustering resolutions, ranging from 0.2 to 1.5, to determine the optimal number of cell clusters. In order to mitigate the impact of batch effects in datasets containing many samples, the RunHarmony [29] function was employed for batch correction. In the end, the data underwent dimensionality reduction and visualization using the RunTSNE and RunUMAP functions, allowing for the representation of the data in lower-dimensional spaces.

### Cell cycle analysis

The Tricycle [30] R Package version 1.8.0 was utilized to infer the cell cycle of single-cell sequencing and visualized the results. The process began by converting the normalized single-cell data into a SingleCellExperiment object using the as.SingleCellExperiment function. The routines estimate_cycle_position and estimate_Schwabe_stage were used thereafter to determine the cell's position and its stage, respectively, allowing for future visualization.

### CellChat analysis

The investigation was conducted using the CellChat R package (version 1.6.3) [31]. At first, a CellChat data entity that can be recognized was created. The analysis utilized the CellChatDB.mouse and CellChatDB.human receptor interaction databases. The existing functionalities of identifyOverExpressedGenes and identifyOverExpressedInteractions were utilized to discover genes that exhibited higher expression levels and their accompanying interactions. The communication probability was determined by utilizing the computeCommunProb, filterCommunication, and computeCommunProbPathway functions. The netAnalysis_contribution function was applied to calculate the contribution of each ligand-receptor pair to the whole signaling pathway. The extractEnrichedLR function was utilized to retrieve all significant ligand-receptor pairing and their corresponding signaling genes for a certain signaling pathway. The netVisual_bubble tool was used to graphically depict the significant ligand-receptor interactions among the cells of interest.

### Consensus non-negative matrix factorization (cNMF) analysis

The gene expression data of 32 HCC samples were analyzed. The consensus Non-negative Matrix Factorization [32] (cNMF) method was employed to identify potential expression programs within each tumor sample. This analysis revealed the presence of six unique programs per sample, resulting in a total of 192 intratumoral expression programs. After calculating the Pearson correlation coefficients for these programs, they were categorized into corresponding meta-programs. By manually curating the data, we conducted enrichment analysis to the thirty genes with the highest variability from the cNMF results. This analysis was performed across sixty-four cancer case pathways, resulting in the identification of six common meta-programs.

### Single sample gene set enrichment analysis (ssGSEA)

After annotating each cluster in the single-cell dataset, the Findallmaker function was employed to find genes that exhibit significant variability within immune cell subpopulations. Following that, a study of immune infiltration was performed using ssGSEA. This study involved a group of 375 tumor samples and 50 normal tissue samples obtained from the TCGA-LIHC dataset. The ssGSEA analysis was conducted using the R package GSVA [33].

### Consensus clustering analysis

To enhance our comprehension of the immune milieu in HCC, it is crucial to perform a more detailed examination of the correlation between immunological scores and patient survival [34]. ConsensusClusterPlus [35] (version 1.64.0) was utilized to conduct consensus clustering of TCGA-LIHC patients (conducted using the “hc” algorithm and “Pearson” distance). The cluster number k ranged from 2 to 10, and the optimal k was determined based on the cumulative distribution function (CDF) and area under the curve (AUC). Leading to their categorization into distinct clusters based on their prior immune scores (derived from ssGSEA). Subsequently, survival curves (Kaplan–Meier curves) were conducted between immune subtypes for differences.

### Trajectory analysis

A Monocle object was created from the processed Seurat object to do pseudotime analysis on a specific subset of macrophages, obtained using the subset function. The estimatedSizeFactors were utilized to standardize the data. The DDRTree technique was utilized to do dimensionality reduction analysis. The orderCells function was employed to deduce the developmental trajectory in the pseudotime analysis [36]. In the ensuing inquiry, the determination of cell differentiation potential is examined concurrently utilizing cytoTRACE, after the acquisition of Monocle findings [37]. According to the results of cytoTRACE, cell subsets that had reduced ability to differentiate (referred to as less.diff) were identified as the initial point of the trajectory, while those with increased ability to differentiate (referred to as more.diff) were considered as the endpoint of the differentiation trajectory. Moreover, the cell subsets that show increased differentiation provide confirmation and refinement to the trajectory direction described by Monocle. The Slingshot algorithm [38] was subsequently utilized to examine the differentiation pathway of macrophages in human single-cell data. The getLineages function was used concurrently to demonstrate the direction of differentiation, in order to address the challenges posed by widely dispersed trajectories of cell subpopulations.

### Spatial transcriptome analysis

The evaluation of the cell signature gene set was performed using the AddModuleScore function in the Seurat R package as part of the analysis. The enrichment score can be visually depicted on a spatial transcriptomic landscape. The data preprocessing step entailed employing the SCTransform function [39], followed by a subsequent principal component analysis (PCA). The ElbowPlot function was utilized to ascertain the most optimal number of principal components. Furthermore, the cell score data obtained from the four spatial transcriptomics datasets underwent a one-way analysis of variance (ANOVA) using GraphPad Prism 9 for statistical analysis. To evaluate the spatial colocalization of ligand-receptor pairs, it was imperative to collect the spatial coordinates and associated expression levels for each ligand and receptor at all examined locations. Subsequently, the subset of spots that belong to the top quintile, comprising those with the highest levels of expression, was selected for further investigation. The assessment of the nearest neighbor [40] was then conducted by evaluating the six nearest adjacent locations and implementing this procedure using the R package RANN. The procedure of normalizing data expression level was performed to guarantee that the data is standardized and can be compared consistently throughout the entire dataset.

### Scoring macrophages using subset-specific signature genes

An evaluation was performed on each macrophage subset utilizing the signature genes derived from specific macrophage subpopulations in order to determine their functional preferences. More precisely, genes that were characteristic of Clec4f+ Macrophage (Kupfer cells) and other macrophage subtypes were identified based on their absolute log2FC value being greater than 0.3 and their adjusted P-value being less than 0.05, when comparing the transcriptome of Clec4f+ Macrophage to other macrophages. The signature gene score for each cell was calculated using the formula [41]:

$$\mathop \sum \limits_{signature\,genes} E_{i,j} /\left( {\mathop \sum \limits_{all\,genes} E_{i,j} \times N} \right)$$

(j represents the expression level of the gene, i represents the cell, and N represents the number of signature genes).

### Clinical modeling

The identified Scissor Positive cell Correlated Genes (SPCG) genes were subjected to Univariate Cox regression, lasso regression, and multivariate Cox regression analysis to obtain independent predictive genes. The risk score for each patient was calculated using the following formula:

$${\text{Risk score}} = \mathop \sum \limits_{k = 1}^{n} \left( {EXP_{i} {*}COEF_{i} } \right)$$

(SC represents the risk score, EXPi represents the expression level of each gene, and COEFi represents the regression coefficient for each gene).

### Functional enrichment analysis

Gene Ontology (GO), Kyoto Encyclopedia of Genes and Genomes (KEGG), and gene-set enrichment analysis (GSEA) were executed using the R package clusterProfiler [42] and ClusterGVis (GitHub-junjunlab/ClusterGVis: One-step to Cluster and Visualize Gene Expression Matrix). The results from both the GO and KEGG enrichment investigations were ranked according to their level of significance, and only the top 20 most significant results were retained. The c5.go.symbols.gmt gene set from the Molecular Signatures Database (MSigDB) (GSEA | MSigDB (gsea-msigdb.org)) was selected as the target in the GSEA. The visualization and analysis operations were performed utilizing the efficient online platform SangerBox [43].

### RNA isolation and real-time quantitative PCR (RT-qPCR)

The expression of three genes (MED8, YBX1, and KPNA2) in tissue samples was verified using RT-qPCR. The liver's RNA was extracted using Trizol reagent (Vazyme, Nanjing, China) following the manufacturer's procedure, and then reverse transcribed using the HiScript® II Q RT SuperMix for qPCR kit (Vazyme, Nanjing, China). The LightCycler®96 Real-Time PCR Detection System (Roche, Basel, Switzerland) and the ChamQ SYBR qPCR Master Mix (Vazyme, Nanjing, China) were used to conduct RT-qPCR. The primer sequences can be found in Additional file 1: Table S1. The 2^−ΔΔCT^ method was employed to ascertain ploidy alterations at the gene level, with GAPDH serving as the normalization gene. The PCR reaction was conducted three times. The Ethics Committee of the Anhui University of Chinese Medicine (AHUCM-mouse-2023121) granted approval for experiments conducted on animal samples.

### Statistical analysis

The statistical analyses were performed using R software (version 4.2.2), Python (version 3.7), and GraphPad Prism 9. Correlation analysis was performed using Spearman rank correlation or Pearson correlation. Various statistical approaches, including the Wilcoxon test, Student's *t*-test, and one-way analysis of variance (ANOVA), were used to analyze differences across groups.

## Results

### Single-cell landscape of NASH and HCC

**Fig. 2:**
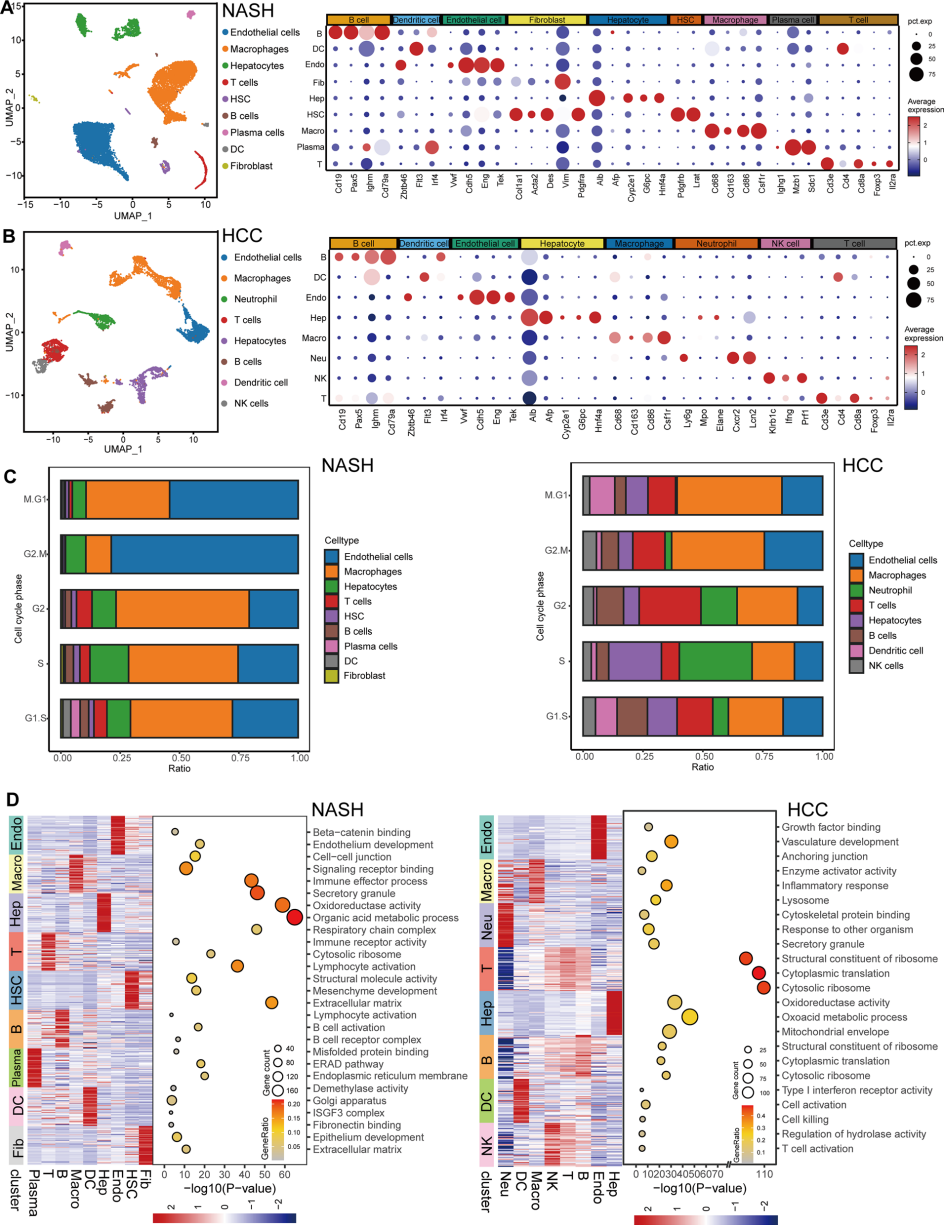
Overview of single-cell transcriptome in NASH and HCC. **A** Cluster annotation and cell type identification of the single-cell dataset in NASH (GSE129516) was generated using UMAP plot(left), and a bubble diagram illustrates the characteristics of each cell marker of NASH (right), the dot size indicates the fraction of expressing cells, and dots are colored based on average expression levels. **B** Cluster annotation and cell type identification of the single-cell dataset in HCC (GSE142868) was generated using UMAP plot(left), and a bubble diagram illustrates the characteristics of each cell marker of HCC (right), the dot size indicates the fraction of expressing cells, and dots are colored based on average expression levels. **C** The proportion of single cells across the cell cycle phases in the NASH (GSE129516, left) and HCC (GSE142868, right) dataset. **D** Heatmap showing expression signatures of the top 50 specifically expressed genes in each cell type of NASH (GSE129516, left) and HCC (GSE142868, right). The value for each gene is row-scaled Z score, representative GO terms are displayed on the right side of the heatmap. (*B* B cell, *DC* Dendritic cell, *Endo* Endothelial cell, *Fib* Fibroblast, *Hep* Hepatocyte, *HSC* Hepatic stellate cell, *Macro* Macrophage, *Plasma* Plasma cell, *T* T cell, *Neu* Neutrophil, *NK* Natural killer cell)

We identified nine different cellular identities by examining 13,580 single-cell transcripts in the NASH dataset (GSE129516) as part of our ongoing work. Furthermore, we visually depicted the primary cell markers for each subgroup using bubble charts, as illustrated in Fig. 2A. Moreover, the HCC dataset yielded a total of 6363 single-cell transcripts, which allowed for the discernment of eight unique cellular identities. The unique features of these specialized cells were illustrated using bubble maps, as shown in Fig. 2B Following cellular characterization, we conducted a comparative analysis of the cell cycle in single-cell datasets of both NASH (GSE129516) and HCC (GSE142868). The NASH dataset displayed a reduced ratio of cell types in active mitotic phases. By contrast, the HCC dataset showed a more diverse distribution of cells across different phases of the cell cycle, particularly among macrophages, T cells, and hepatocytes, suggesting an elevated level of cell proliferation (Fig. 2C). In addition, our research showed that endothelial cells and macrophages were present in large quantities during all stages of the cell cycle in the HCC group, and this pattern was also observed in the NASH group. Significantly, the endothelial cells exhibited a pronounced predominance in the G2/M phase.

Subsequently, we performed a GO analysis on the genes that were differentially expressed (DEGs). We then examined the Hallmark pathway for each cell cluster to enhance our comprehension of the biological processes linked to each cell cluster (Fig. 2D and Additional file 2: Fig. S1). The current study unveiled comparable biological functions in both NASH and HCC. The cytosolic ribosome pathway showed substantial activity in T cells, suggesting a heightened level of cellular activity in both conditions. Concurrently, macrophages displayed a diverse array of immune-related biological processes, suggesting the intricate immunological milieu in the NASH-HCC. To summarize, the results obtained from this study highlight the substantial contribution of immune cells in the progression of NASH and HCC.

### NASH and HCC, the MIF signaling pathway plays a role in hepatocyte-macrophage interactions through intercellular signaling

**Fig. 3:**
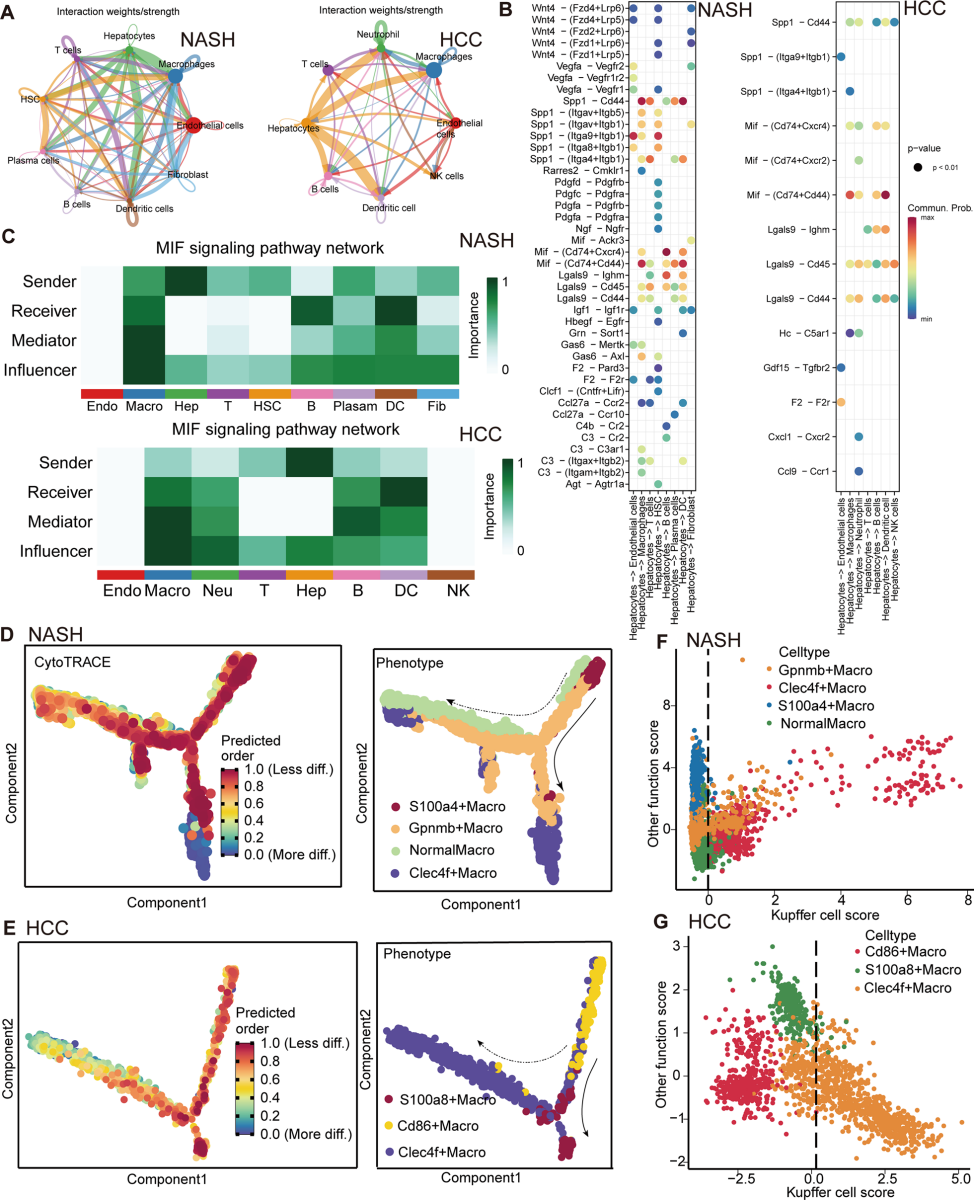
CellChat and Monocle analysis of macrophage subpopulations. **A** The circle diagram illustrates the signal crosstalk of all cells in the NASH dataset (GSE129516, left) and HCC dataset (GSE142868, right), with the thickness representing the signal strength. **B** All the significant ligand-receptor pairs that contribute to the signaling transmission from hepatocytes to other cell types. Dots represent the contribution of each receptor pair in signals emitted by hepatocytes toward various cells in the NASH dataset (GSE129516, left), and HCC dataset (GSE142868, right). Dot size indicates significance, while color shade represents the magnitude of contribution. Darker shades, particularly red, indicate a higher contribution, while lighter shades indicate a lower contribution. **C** Heatmap shows the relative importance of each cell group based on the computed four network centrality measures of the MIF signaling network in NASH (GSE129516, up) and HCC dataset (GSE142868, down). **D** cytoTRACE (left) and monocle2 (right) are combined to predict the differentiation potential and direction of macrophage subtypes in the NASH (GSE129516) dataset; more.diff means higher differentiation potential, and as the end point of monocle, the less.diff is considered the starting point of monocle. **E** cytoTRACE (left) and monocle2 (right) are combined to predict the differentiation potential and direction of macrophage subtypes in the HCC (GSE142868) dataset; more.diff means higher differentiation potential, and as the end point of monocle, the less.diff is considered the starting point of monocle. **F** Stratification of macrophage transcriptomes by scores generated from signature genes of four macrophage subsets in NASH dataset (GSE129516). **G** Stratification of macrophage transcriptomes by scores generated from signature genes of three macrophage subsets in the HCC dataset (GSE142868). (NormalMacro: classical macrophages.)

The CellChat R package was utilized to conduct a thorough investigation of two separate single-cell datasets (GSE129516, GSE142868), leading to an improved understanding of the intercellular signaling pathways across different cell types. This method facilitated the analysis of intricate intercellular communication networks and allowed the assessment of specific ligand-receptor interactions utilizing a collection of cellular communications. The analysis revealed robust intercellular contact between hepatocytes and immune cells, particularly myeloid cells, in both NASH and HCC (Fig. 3A).

MIF has been found to have a role in various crucial biological processes, including inflammatory and immunological responses, cell proliferation controlled by growth factors, cell cycle regulation, angiogenesis, and tumorigenesis. The inflammatory function of MIF is evidenced by its interaction with a receptor complex composed of CD74, CD44, CXCR2, and CXCR4. Our analysis revealed a notable increase in the signal strength of the MIF-(CD74/CD44) ligand-receptor pair, originating from hepatocytes and targeting macrophages in both NASH and HCC, as depicted in Fig. 3B. Consequently, we performed an extensive examination of various communication patterns within the MIF signaling pathway, with a specific emphasis on the interactions between macrophages and hepatocytes. Consistent with our original findings, macrophages predominantly received intercellular signals, while hepatocytes primarily emitted these signals. The discovered interaction pattern exhibited a high level of consistency in both of the analyzed disease states, as depicted in Fig. 3C. Furthermore, the MIF signaling pathway had been recognized as the principal means of communication, serving as both a key emitter and receiver of signals. The consistency of this pattern was confirmed in both single-cell datasets, as illustrated in Additional file 2: Fig. S2A–D. These data emphasize the significance of the MIF signaling pathway as the primary ligand-receptor pair that enables interactions between hepatocytes and macrophages, thus proving its substantial involvement in the progression of both NASH and HCC.

### NASH and HCC macrophage subpopulations: differentiation, infiltration, and disease progression-related consequences

To gain a more thorough comprehension of the involvement of macrophages in the progression of NASH and HCC, we employed the subset function to separate particular subgroups of macrophages from two distinct single-cell datasets (GSE129516 and GSE142868). We applied a dimensionality reduction technique to visually portray the different subgroups of macrophages. Additionally, we used proven single-cell analytic tools to investigate macrophage markers that are already known (Additional file 2: Fig. S3A, B).

CytoTRACE analysis was employed to determine the differentiation capacity and establish the pseudotemporal trajectory of cellular progression. This analysis demonstrated that Kupffer cells, namely Clec4f+ macrophage, had the highest degree of differentiation potential, as shown in Additional file 2: Fig. S3C, D.

Furthermore, under the framework of Monocle-based pseudotime evaluation, the Clec4f+ macrophage was conjectured as the final stage of differentiation. We observed that the Gpnmb+ macrophage, which had similarities with tumor-associated macrophages and was associated with poor prognostic implications [44], showed a remarkable ability to differentiate (Fig. 3D).

Under the context of HCC, we had discovered three clearly defined subsets of macrophage. These subsets consist of Cd86+ macrophages, which were characterized by their proinflammatory properties, S100a8+ macrophages, which were associated with proinflammatory [45], and Clec4f+ macrophages, which were specifically recognized as Kupffer cells [46]. The study’s investigations on HCC shown that Clec4f+ macrophages had a higher degree of differentiation ability in comparison to other cell types. Subsequently, we performed pseudotime analysis on the HCC macrophage subgroups using Monocle. The expression of Cd86+ macrophages and S100a8+ macrophages consistently declined as pseudotime progressed, but the abundance and proliferation of Clec4f+ macrophages increased during this developmental phase (Fig. 3E).

By directly stratifying macrophage subsets, it was observed that Gpnmb+ macrophages and Clec4f+ macrophages exhibited a stronger preference for Kupffer cells in NASH. The results were also observed in HCC, as shown in Fig. 3F, G. It is worth mentioning that these specific types of cells were located at the end stage of differentiation, as indicated by cytoTRACE and Monocel. This observed consistency provides support for the inference that the Kupffer cells of the liver may be populated by macrophages originating from other subtypes [47]. The discovered phenomenon displayed a notable degree of uniformity amongthe macrophage subpopulations in NASH and HCC.

The role of immune cells in the development of NASH and HCC is clearly evident [14, 44], emphasizing the need to determine the specific immune cell type involved in these disorders. For this study, we obtained comprehensive datasets including annotated single-cell data and bulk transcriptome data for both disease states. Gene sets were generated for each cell population, enabling a comprehensive analysis of cell infiltration using the ssGSEA algorithm. In addition, the results from CIBERSORTX were referenced. The NASH group highlighted the differences between macrophages and hepatic stellate cells (HSCs) (Additional file 2: Fig. S4A). Additionally, the CIBERSORTX analysis showed that macrophages were specifically enriched in the disease group (Additional file 2: Fig. S4B). Conversely, significant differences in different types of immune cells were noticed within the HCC group. The study revealed a significant difference in macrophage infiltration between the HCC group and the normal group, with a much higher level reported in theHCC group (Additional file 2: Fig. S4C). The results obtained from the CIBERSORTX analysis showed a clear increase in the number of macrophages in the illness group (Additional file 2: Fig. S4D).

To further support our findings, we performed differential gene analysis on the RNA-seq data (Additional file 2: Fig. S4E) and conducted GSEA using the single-cell Gene Matrix Transposed (GMT) file format that we had previously collected. Notably, there were clear differences found specifically in macrophages (Additional file 2: Fig. S4F) and HSCs (Additional file 2: Fig. S4G) within the NASH group. The macrophages displayed notable distinctions exclusively within the HCC group, as evidenced by unequivocal consensus (Additional file 2: Fig. S4H). The data consistently confirmed our first observations. The findings of this study provided compelling evidence to support the pivotal function of macrophages in promoting the interaction between NASH and HCC, suggesting their potential as a crucial link that connects the two disorders.

### HCC patient cellular subpopulations, macrophage pathology, and intratumoral heterogeneity

**Fig. 4:**
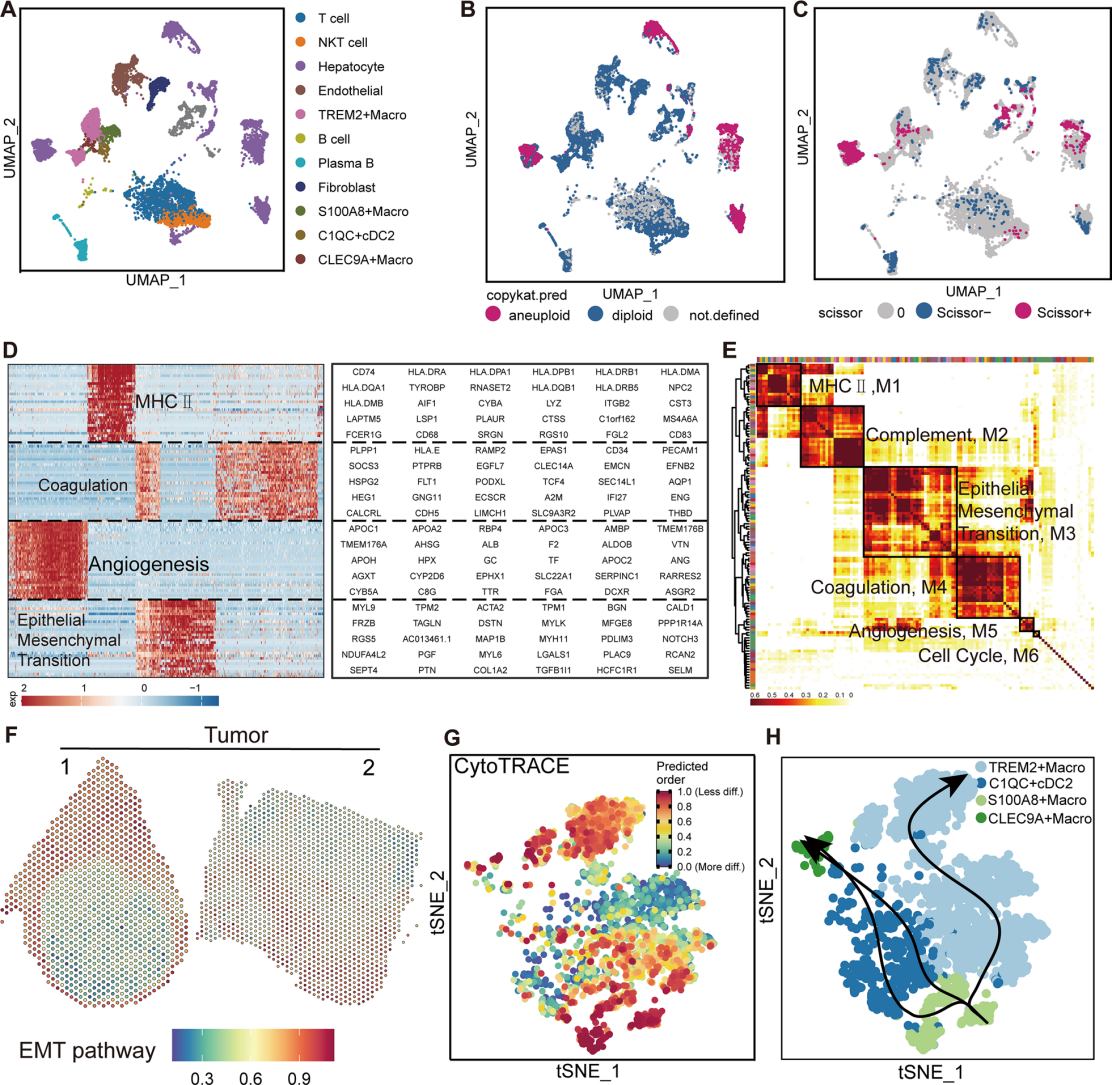
Summary of single-cell sequencing data from HCC patients and subtypes of macrophages.** A** Of 32 samples with HCC single-celled sequencing data analysis, using the UMAP plot shows 11 cell clusters, colored by cell cluster. **B** Employing the CopyKAT algorithm to determine benign and malignant cells, most of the hepatocytes were inferred as malignant cells. **C** UMAP visualization of Scissor algorithm-identified Scissor-positive and Scissor-negative cells, Hepatocytes, Macrophages, and NK T cells are considered scissor-positive cells. **D** Heatmap shows differentially expressed genes (rows) identified by cNMF clustered by their expression across single cells (columns) from a representative patient. The gene clusters reveal intratumoral programs that are differentially expressed. The corresponding gene signatures are indicated (right). **E** Unbiased Clustering reveals six programs in HCC, Heatmap depicts pairwise correlations of 64 intratumoral programs derived from 32 tumor samples. **F** GSVA was used to compute the EMT pathway for spatial localization at the tumor-normal interface, with red representing the highest intensity of each SPOT expression and blue representing the lowest. **G** Using cytoTRACE to predict the differentiation potential and direction of macrophage subtypes; more.diff means higher differentiation potential, and as the end point of Slingshot, the less.diff is considered the starting point of Slingshot. **H** Using Slingshot to analyze macrophage subgroup trajectory of differentiation, relies on the results of cytoTRACE, S100A8+ Macro as the starting point, and the rest of the cell type as the destination. (cDC: conventional Dendritic cell)

In the preceding section, our study centered on examining the relationship between NASH and HCC in mice models. Following that, a comprehensive investigation was conducted by analyzing single-cell samples obtained from individuals with HCC (GSE151530). 32 specimens collected from individuals with HCC were subjected to a rigorous cell filtration process to assess their suitability for a thorough analysis. Through this method, 11 different cell clusters were identified, as illustrated in Fig. 4A. Accurately characterizing cellular subpopulations associated with illnesses requires the essential incorporation of T cell subsets and myeloid lineage cells. Concurrently, a comprehensive validation of cellular markers was conducted to guarantee accurate identification of cell types (Additional file 2: Fig. S5A, B). We employed the CopyKAT algorithm to predict the presence of malignant cell populations in these cohorts. Consistent with our predictions, a significant proportion of hepatocytes were identified as cancerous cells, which confirms the reliability of our initial annotation (Fig. 4B).

In order to confirm the pathogenic significance of macrophages in HCC, the single-cell data was combined with RNA-seq data from 375 patients in the TCGA dataset. The implementation of the Scissor algorithm facilitated the identification of pivotal pathogenic cell subgroups, resulting in the accurate inference of malignant hepatocytes, macrophages and NK T cells. (Fig. 4C).

Due to the large number of samples, it was determined that traditional functional analyses were not sufficient. As a result, the cNMF methodology was adopted to better understand the intratumoral heterogeneity within HCC. The samples were found to have distinct expression programs, which were characterized by unique combinations of gene modules that showed high expression levels in specific tumor cases. For instance, in a representatively sample of patients with HCC that accurately reflects the overall population, four well defined clusters of gene signatures were discovered (Fig. 4D). Among the 32 tumor specimens, a total of 62 expression programs were discovered (Additional file 1: Table S2). The highly expressed consistency of signatures from different tumors reflects a common pattern of expression heterogeneity within tumors. Pearson correlation analysis was performed on these expression programs, and then they were integrated into a single module. We manually identified six co-expression programs in our investigation, as shown in Fig. 4E and described in Additional file 1: Table S3. Program 1 (M1) was distinguished by the presence existence of major histocompatibility complex (MHC) class II molecules, specifically CD74 and HLA-DMA. The second program (M2) exhibited a unique pattern of PLAUR and FCER1G expression, indicating the elimination of immune complexes and the presence of inflammation in the surrounding environment. The third program (M3) was linked to the presence of TPM1 and MYL9, and it exhibited an association with the incidence of epithelial-mesenchymal transition (EMT). M4, distinguished by the presence of PECAM1 and THBD, was linked to the coagulatory function, a frequently reported issue in persons with HCC. The M5 gene cluster included CD34 and EFNB2, which functioned as indicators of angiogenic activity. The sixth program exhibited a robust correlation with cell cycle processes, as seen by the expression of CHAF1B and CDC45.

Our study revealed a noteworthy augmentation in the enrichment of the EMT pathway at the peritumoral margin compared to the intratumoral region (Fig. 4F). This discovery was significant as it further reinforces the documented correlation between the EMT process and TAMs. Emphasizing the need for a focused examination of this issue. In addition, we performed a comparative analysis to evaluate the capacity for differentiation of three more precisely characterized subsets of macrophages, along with a percentage of dendritic cells. Our findings demonstrated a higher prevalence of TREM2+ macrophages (Fig. 4G, H), which was also associated with a worse prognosis [48].

### HCC patient macrophage infiltration and MIF signaling spatial characterization

**Fig. 5:**
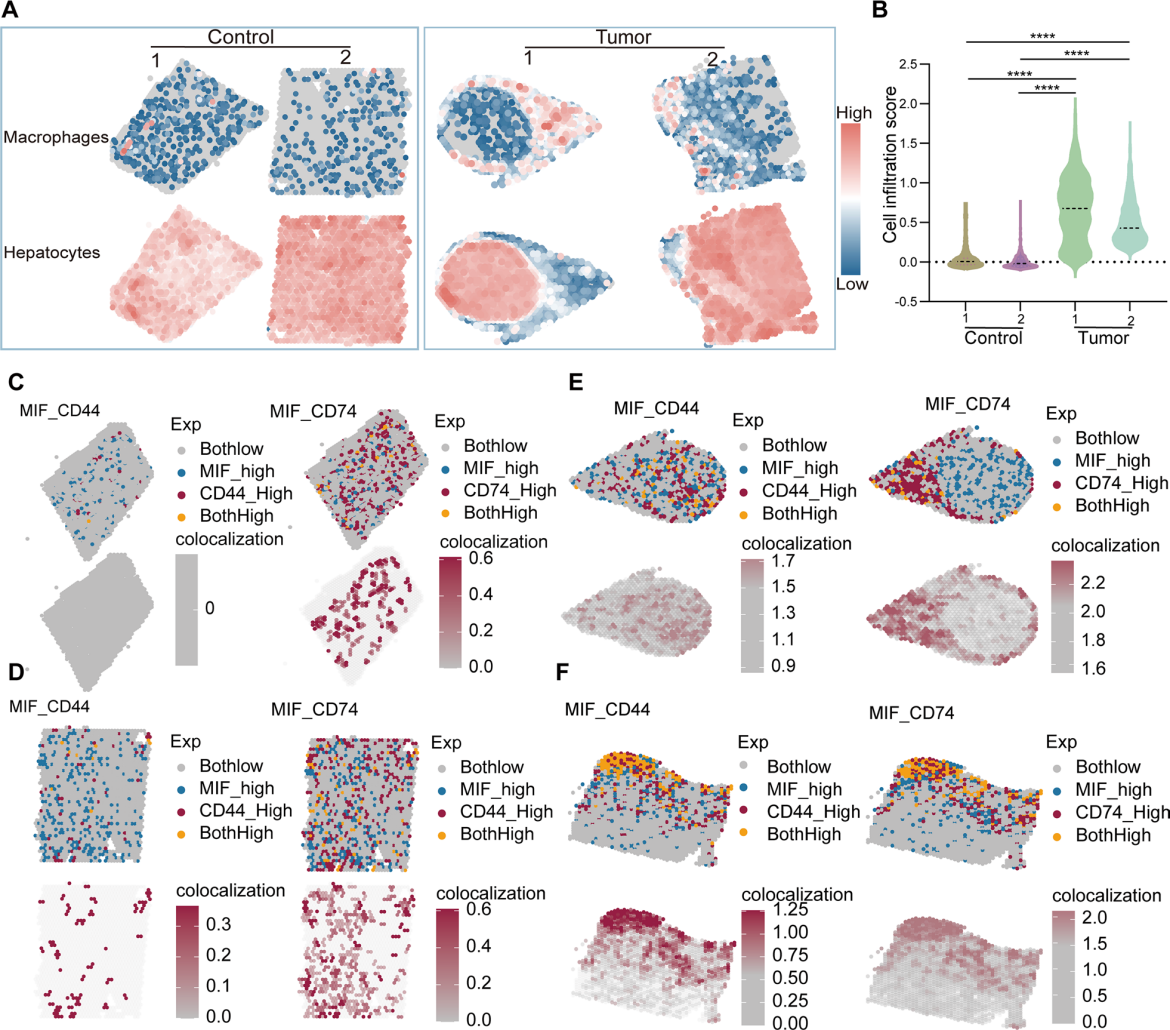
Spatial colocalization of macrophages, hepatocytes, as well as MIF, CD74, and CD44. **A** Mapping of the enrichment scores of macrophages and hepatocytes onto a single-cell spatial transcriptome map, reflecting their infiltration in both tumor and normal tissues, by using the Addmodulescore function. **B** One-way analysis of variance (ANOVA) was performed to test the infiltration scores of macrophages in the four spatial transcriptome atlases. Significance is denoted as follows: *P < 0.05; **P < 0.01; ***P < 0.001; ****P < 0.0001. **C**–**F** Spatial colocalization of MIF with CD44 and CD74 in the Control1 group (**C**), Control2 group (**D**), Tumor1 group (**E**), and Tumor2 group (**F**). Exp means the relative expression of the ligand and the receptor; colocalization means the interaction strength

To accurately describe the spatial interaction between HCC cells and the associated macrophage populations, we utilized extensive single-cell datasets acquired from HCC samples (GSE151530). We were able to uncover distinct gene signatures that are specific to hepatocytes and macrophages. The gene signatures were matched with spatial transcriptome profiles using the AddModuleScore program, enabling precise mapping of cellular distributions. The current methodology demonstrated a distinct pattern of macrophage infiltration in two HCC specimens, which was conspicuously absent in adjacent non-tumorous tissue samples (Fig. 5A). These findings aligned with the expected EMT events that were frequently linked to the development of tumors. In addition, the results were corroborated by a variance analysis performed on the infiltration scores derived from spatial transcriptomic data (Fig. 5B).

Subsequent research was conducted utilizing CellChat to authenticate the involvement of MIF signaling in HCC. Consistent with previous observations, it is shown that hepatocytes predominantly exhibited MIF signaling, while macrophages demonstrated the highest degree of responsiveness (Additional file 2: Fig. S6A). Simultaneously, it was revealed that the signaling crosstalk between hepatocytes and macrophages involved the participation of important ligand-receptors, namely CD44–CD74 (Additional file 2: Fig. S6B). Ultimately, our data offer additional proof that MIF signaling played a crucial role in intercellular communication (Additional file 2: Fig. S6C, D). The results aligned with the findings of our earlier investigation.

Building upon our previous emphasis, we had conducted additional research on the spatial dynamics of the MIF signaling pathway, specifically its interaction with the CD44–CD74 ligand receptor complex. The subsequent examination demonstrated an increased spatial co-localization between MIF-CD44 and MIF-CD74 within the HCC tissues, with a particularly noticeable effect around the edges of the tumor. In sharp contrast, the interaction between the ligand-receptor interplay in the peritumoral areas was significantly weaker, showing minimal evidence of colocalization (Fig. 5C–F). The aforementioned observations significantly enhance our comprehension of the immunological microenvironment in HCC.

### HCC immunological subgroups and pathway enrichment

**Fig. 6:**
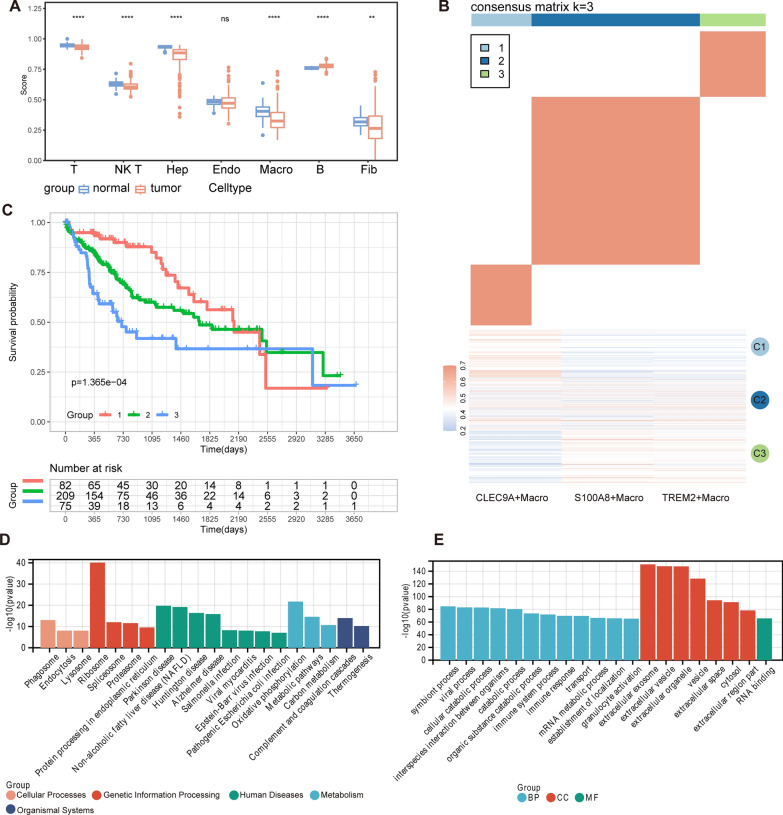
Immune differences in populations and functional enrichment. **A** Immune cell infiltration differential analysis based on ssGSEA algorithm. Significance is denoted as follows: ns nonsignificance; *P < 0.05; **P < 0.01; ***P < 0.001; ****P < 0.0001. **B** Identification of immune subtypes and expression signature modules in TCGA-LIHC performed by ConsensusClusterPlus, Consensus clustering matrix for k = 3. **C** Kaplan–Meier survival analysis based on immune subtypes in the TCGA-LIHC cohort. **D**, **E** KEGG (D) and GO (E) enrichment analysis display the top 20 most significant results

This study conducted immune infiltration assessments by analyzing RNA-sequencing data obtained from HCC patients in the TCGA database. A strong correlation was seen between the results of the ssGSEA and the Scissor algorithm, specifically in relation to the identification of macrophages (Fig. 6A). This discovery suggested the existence of consistent immunological fluctuations.

To enhance our comprehension of the diversity in immune response across patients with HCC, we performed stratified clustering analysis on the immune infiltration profiles obtained from prior studies. Through this investigation, three distinct immunological subgroups had been identified, each demonstrating notable variations. The CLEC9A+ macrophage population was shown to be more abundant in the C1 immunological subgroup. On the other hand, it was demonstrated that all three identified macrophage subsets were found to be more prevalent within the C2 subtype (Fig. 6B). Survival analyses applied to each immunological subtype yielded distinct predictive results, in line with our initial predictions (Fig. 6C).

The identification of characteristic genes in scissor-positive cells was performed by extra analyses using the FindAllMarkers function of the Seurat R package. The gene collection, consisting of 3560 genes (Additional file 1: Table S4), underwent GO and KEGG enrichment analysis. The analysis mainly focused on the top 20 enrichments. The analysis of the KEGG enrichment indicated a notable emphasis on pathways related to NAFLD, specifically within the Human Disease (HD) cluster. Furthermore, the significance of the ribosome pathway was emphasized, suggesting a higher level of protein biosynthesis and cellular activity (Fig. 6D). The GO enrichment analysis revealed that categories such as extracellular exosomes, organelles, and vesicles were highly prevalent. This emphasized the crucial significance of intercellular communication in the biological environment being studied (Fig. 6E).

### Identification and validation of HCC prognostic genes

**Fig. 7:**
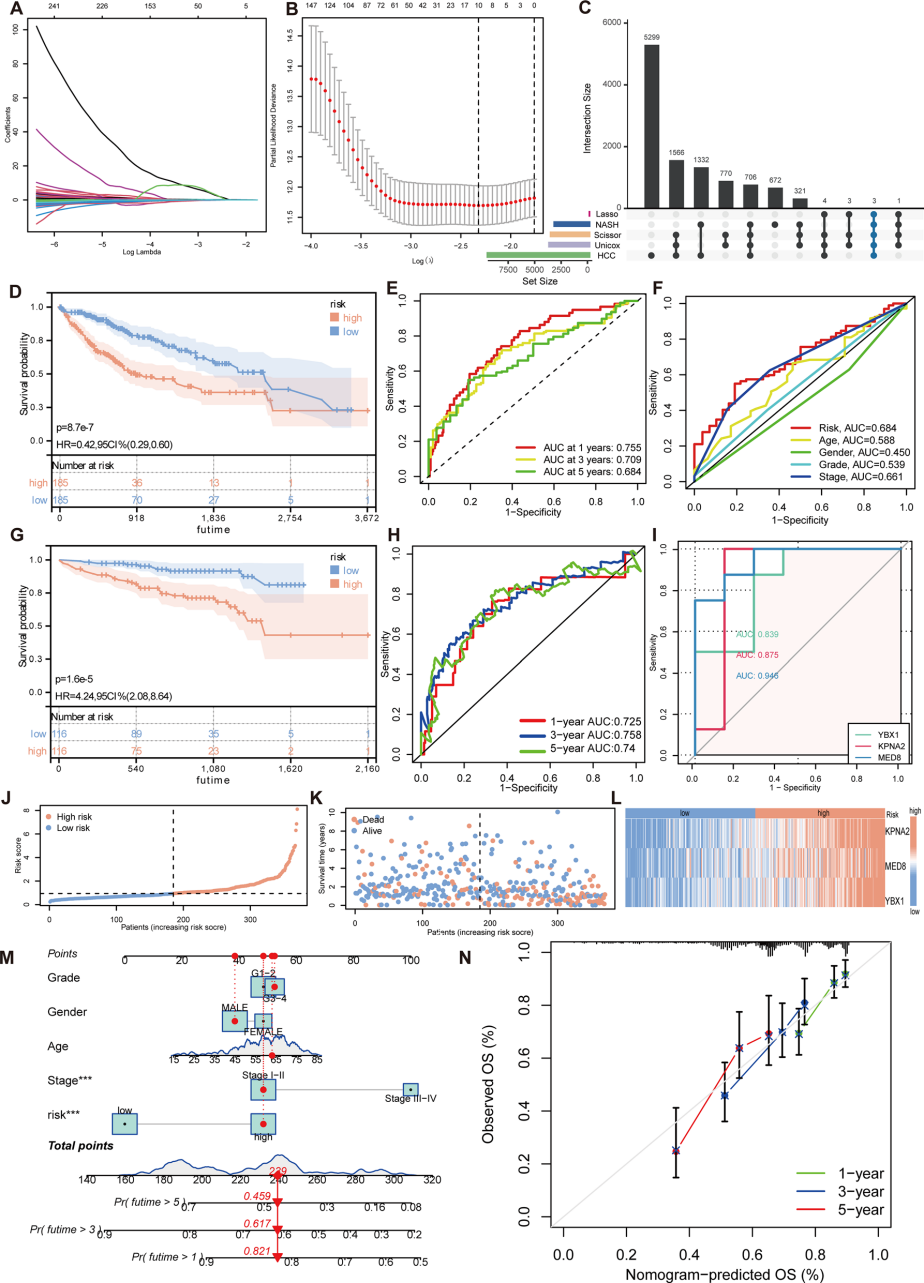
Construction and validation of clinical predictive models. **A** LASSO regression coefficient graph. **B** Partial likelihood deviation of the LASSO coefficient distribution. The two vertical dashed lines represent lambda.min and lambda.1 se. **C** Intersecting gene sets screened under 5 different results, retain KPNA2, YBX1, and MED8. Unicox represent Univariate cox regression. **D** Kaplan‒Meier survival analysis of the HCC patient (TCGA-LIHC) with risk scoring. **E** ROC analysis for predicting the 1/3/5-year survival rate of HCC (TCGA-LIHC). **F** ROC curve analysis for risk scores and other clinicopathological indicators. **G** Kaplan‒Meier survival analysis of HCC patients (ICGC-LIRI-JP) with risk scoring. **H** ROC analysis for predicting the 1/3/5-year survival rate of HCC (ICGC-LIRI-JP). **I** ROC curve of the NASH cohort. **J**, **K** TCGA patients were categorized into high-risk and low-risk groups, based on the median cutoff of the risk score (**J**), and the distribution of risk scores and patient survival between low and high risk (**K**). **L** Heatmap displaying the expression of prognostic genes of the SPCG model in the high- and low-risk groups. **M** Nomogram of risk groupings and clinical characteristics for predicting survival at 1, 3, and 5 years. **N** Calibration curves for testing the agreement between actual and predicted outcomes at 1, 3, and 5 years

A preliminary analysis of patient data from the TCGA-LIHC dataset was performed to improve prediction indicators for HCC. Patients with insufficient clinical information were eliminated, leading to a group of 370 individuals. We utilized the Scissor-positive cell gene set for univariate Cox regression analysis. The approach enabled us to identify a specific group of 3,373 genes that displayed substantial associations with survival outcomes (Additional file 1: Table S5). By employing a lasso regression, we were able to identify 10 prognostic genes from the provided array (Additional file 1: Table S6; Fig. 7A, B). Utilizing lasso regression results, the differential expression analysis revealed that the genes YBX1, MED8, and KPNA2 exhibited diagnostic significance in both NASH and HCC (Fig. 7C).

Expanding on this study, a multivariable Cox regression model was employed to incorporate factors such as pathological grading, sex, age, and stage, along with the expression of the three genes (YBX1, MED8, and KPNA2). This analysis confirmed that these genes served as prognostic markers, as indicated in Additional file 1: Table S7. Risk scores in the TCGA cohort were generated using regression coefficients derived from patient risk equations that relied on gene expression levels. Afterward, the cohort was divided into high- and low-risk groups using a method that involved selecting the median as the cutoff point. Subsequent univariate and multivariable Cox regression studies revealed that both stage and risk scores were determined to be independent prognostic variables. The studies considered variables including age, sex, pathological grade, and pathological stage (Additional file 2: Fig. S7A, B).

The prognostic value of YBX1, MED8, and KPNA2 in the HCC patient cohort was demonstrated by utilizing Kaplan‒Meier survival plots, resulting in encouraging outcomes (Fig. 7D). The diagnostic efficacy of the risk score was confirmed using Receiver Operating Characteristic (ROC) investigations, resulting in Area Under the Curve (AUC) values of 0.755, 0.709, and 0.684 for predicting 1, 3, and 5 years, respectively (Fig. 7E). The pathological staging showed significant diagnostic efficacy, although it did not surpass the previously indicated prognostic genes (Fig. 7F).

The prognostic module’s robustness was confirmed by validation using the ICGC-LIRI-JP liver cancer dataset. The module's diagnostic capability was proven using the ROC curves for 1-, 3-, and 5-year survival. These curves displayed positive outcomes with AUCs of 0.725, 0.758, and 0.74, respectively (Fig. 7G, H). Furthermore, the diagnostic effectiveness of YBX1, MED8, and KPNA2 in the NASH cohort was evaluated using ROC analysis, revealing their exceptional performance with area under the curve (AUC) values of 0.839, 0.875, and 0.946, respectively (Fig. 7I). An analysis of the high-risk and low-risk groups showed a considerably higher mortality rate in the high-risk group (Fig. 7J, K). The heatmaps illustrating the expression levels of the three separate prognostic genes demonstrated higher expression in the high-risk group compared to the low-risk group (Fig. 7L).

### Development and evaluation of the nomogram for predicting prognosis

We had created a comprehensive nomogram that integrates essential clinical and pathological factors, including age, sex, pathological grade, pathological stage, and prognostic risk score. This nomogram aimed to enhance the accuracy of our prognostic model for HCC (Fig. 7M). The nomogram depicted below functions as a tool to facilitate the evaluation of the likelihood of survival for patients with HCC within a span of 1 year, 3 years, and 5 years. The investigation provided confirmation that the stage of disease and the composite risk score exerted significant influence on survival outcomes. It is crucial to incorporate these parameters, together with three significant prognostic genes, in order to improve the precision of survival forecasts in HCC. The calibration curves for survival at 1-year, 3-year, and 5-year intervals demonstrated a strong alignment with the expected survival probability, suggesting a dependable prediction concordance (Fig. 7N). Henceforth, the predictive tool was designated as the SPCG model.

Following the validation of the nomogram, a subsequent analysis was performed to categorize patient survival according to the severity of the sickness stage, using the SPCG model as a framework. The study findings indicated that those categorized as high-risk in stages I–II or stages III–IV exhibited notably elevated death rates in contrast to those categorized as low-risk (Additional file 2: Fig. S7C). Validating data from supplementary clinical parameters, such as age, sex, and pathological grade, verified the reliability and predictive importance of the model (Additional file 2: Fig. S7D–F).

This study not only validated the model but also focused on evaluating the individual prognostic significance of the three identified risk genes. By analyzing a comprehensive immunohistochemical database, the Kaplan‒Meier survival plots clearly showed distinct survival patterns between high and low levels of gene expression. This reaffirmed that these genes could independently serve as prognostic markers (Additional file 2: Fig. S7G).

### Differences in HCC risk groups' tumor mutation burden (TMB), immunological infiltration, and biological pathways

**Fig. 8:**
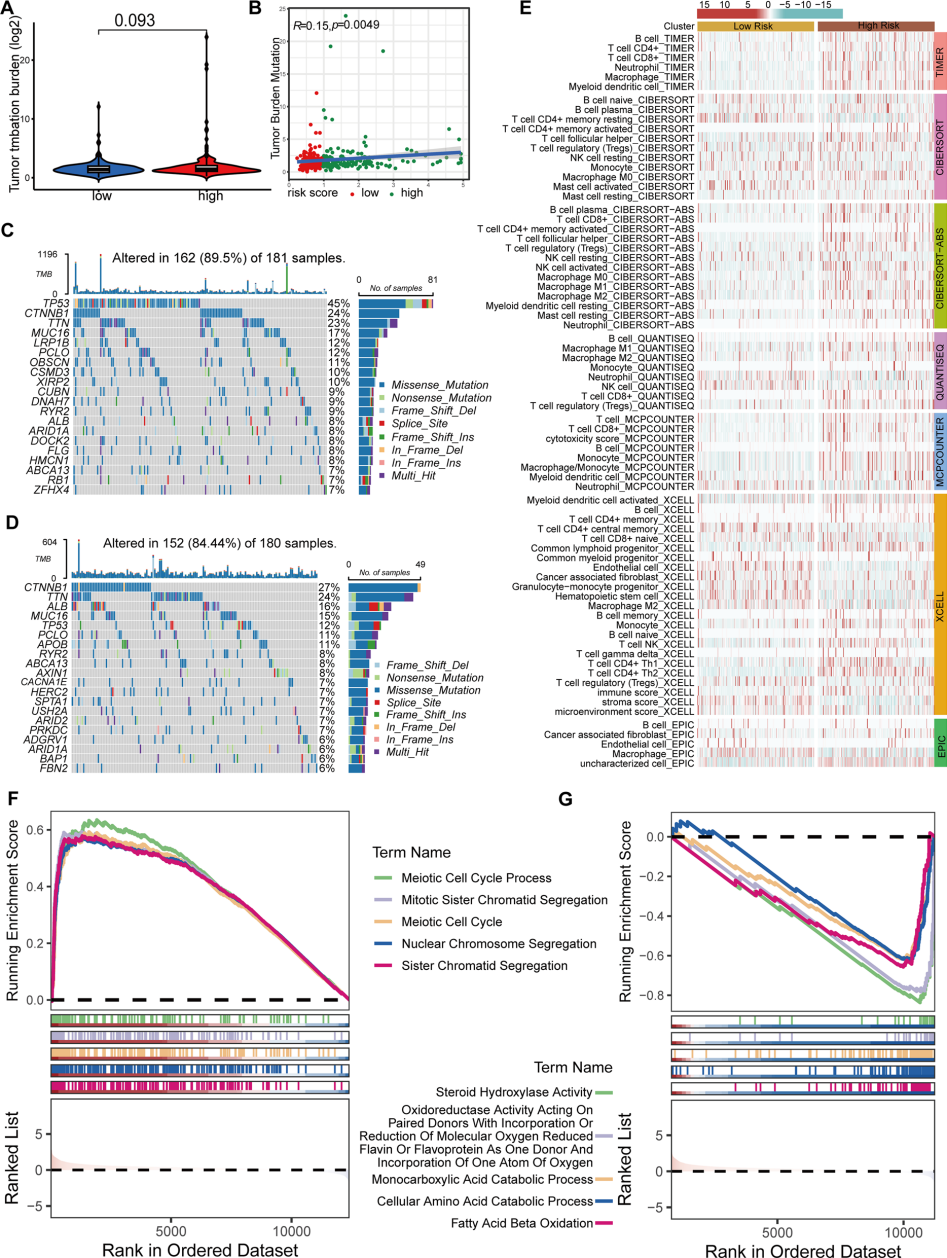
Biological characteristics between high-and low-risk groups. **A** Differences in TMB between high- and low-risk groups. **B** Correlation between TMB scores and risk scores. **C**, **D** Waterfall plot depicting gene mutations in the high- (**C**) and low-risk (**D**) groups. **E** Heatmaps displaying immune cell infiltration analysis results using TIMER, Cibersort, Cibersort-ABS, QUANTISEQ, MCPCOUNTER, XCELL, and EPIC software for the high- and low-risk groups. **F** GSEA for the high- (**F**) and low-risk (**G**) groups, demonstrating the first 5 pathways, NES > 0, activated; NES < 0, inhibited

Within our dataset, the subgroup identified as the high-risk group exhibited a significant TMB of 1196, indicating a substantial degree of genetic mutation. Conversely, the low-risk subgroup demonstrated a TMB of 604, indicating a comparatively lower level of mutations. The discrepancy in TMB scores across the groups did not achieve statistical significance (P = 0.093) (Fig. 8A). The Spearman's rank correlation analysis demonstrated a positive association between the risk score and the TMB score in HCC patients (R = 0.15, P = 0.0049) (Fig. 8B). Analyzed by a waterfall plot, the mutational landscape revealed the frequency of mutations in the top 20 genes. The genes TP53, TTN, and CTNNB1 were shown to have the highest occurrence of mutations. In addition, the high-risk group showed a higher frequency of mutations in these genes compared to the low-risk group (Fig. 8C, D).

We comprehensively assessed the tumor immune microenvironment by utilizing various computer programs such as TIMER, Cibersort, Cibersort-ABS, QUANTISEQ, MCPCOUNTER, XCELL, and EPIC to analyze immune cell infiltration. We employed the Kruskal–Wallis test to determine the immune cell types that showed significant differences in infiltration levels among the groups. Significantly, the immune cells detected by Cibersort-ABS, TIMER, QUANTISEQ, and MCPCOUNTER showed a noticeable difference in infiltration, with a higher presence in the high-risk group. This suggested an immune-active tumor microenvironment. (Fig. 8E).

This study utilized GSEA to elucidate the biological functions that differentiate the high-risk and low-risk groups. The analysis utilized C5: Ontology gene sets obtained from the Molecular Signatures Database (MsigDB). Within the high-risk group, the five pathways that showed the greatest enrichment scores were associated with cellular division activities. These pathways, including the meiotic cell cycle, the meiotic cell cycle process, mitotic sister chromatid segregation, nuclear chromosomal segregation, and sister chromatid segregation, exhibited positive Normalized Enrichment Scores (NES). The observed enrichment highlights an increase in cellular proliferation and mitotic activity among individuals in the high-risk group, which is consistent with the finding from previous GO analyses (Fig. 8F). In contrast, the low-risk group showed notable negative NES in metabolic activities such as cellular amino acid breakdown, fatty acid oxidation, aromatase activity, oxidoreductase activity, and steroid hydroxylase activity. The pathways illustrated in Fig. 8G demonstrated a decrease in metabolic activity, indicating a distinct metabolic phenotype in the low-risk group. This phenotype was characterized by decreased metabolism, secretory capacity, and differentiation.

### The experiment of risk-related genes

**Fig. 9:**
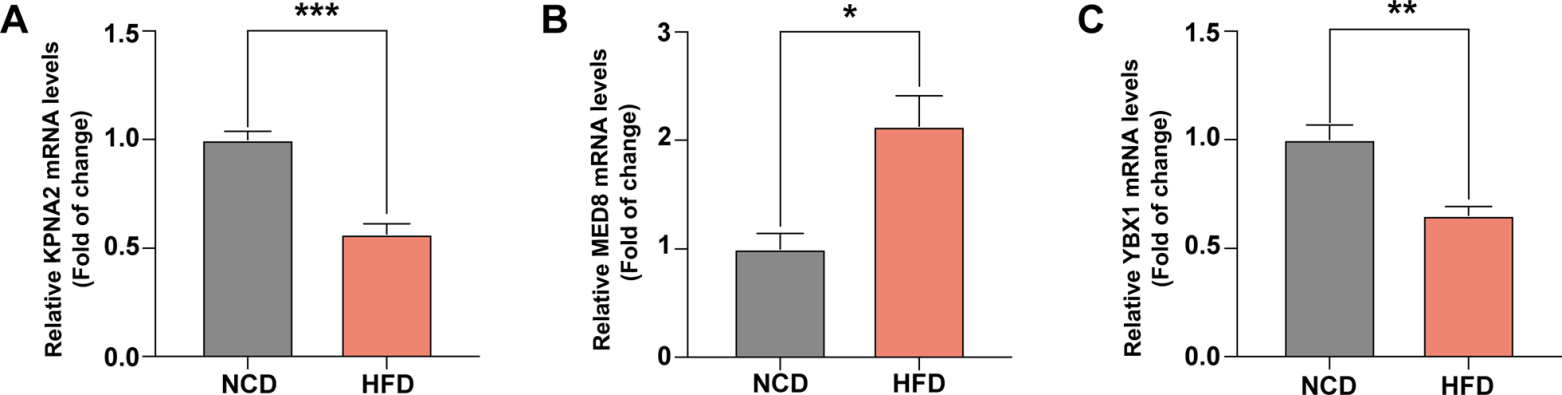
The mRNA expression levels of three prognostic genes in HFD-induced NAFLD mouse model livers and in normal mouse livers were determined by RT-qPCR. **A** KPNA2; **B** MED8; **C** YBX1. P value was calculated by Student’s *t*-test, *P < 0.05, **P < 0.01, ***P < 0.001 compared with the control group

To ascertain the association between these three candidate genes and the advancement of NAFLD to NASH, we employed RT-qPCR to assess the expression of these genes in the liver of a mouse model with NAFLD generated by a high-fat diet (HFD). As demonstrated in Fig. 9, MED8 showed increased expression levels, while KPNA2 and YBX1 showed dramatically decreased expression levels in the liver of mice with NAFLD. Our bioinformatic findings indicated that these genes may function as novel biomarkers for early NASH diagnosis, which was consistent with these distinctions.

## Discussion

NAFLD is a chronic hepatic illness that has the potential to progress to NASH, a more severe stage that can ultimately result in the development of liver cirrhosis and cancer [49]. Based on projections, it is anticipated that approximately 10–20% of patients diagnosed with NAFLD may develop NASH. The delayed identification of NASH sometimes results in the belated discovery of HCC, which in turn leads to an adverse prognosis [50]. Therefore, the quick and accurate detection of NASH and HCC is crucial and has attracted growing attention. The utilization of sequencing technology has facilitated the acquisition of single-cell transcriptome data, which has emerged as the primary approach for identifying markers associated with NASH and HCC [51]. Consequently, contemporary research has placed significant emphasis on examining the cellular landscape of NASH and HCC using single-cell analysis. This includes investigations conducted on animal models [22, 47, 52] and clinical studies involving patients [23, 53]. This study involved a thorough investigation of animal models and clinical samples to examine the relationship between NASH and HCC. In order to accomplish this, we employed single-cell, bulk, and spatial transcriptome data acquired from samples of NASH and HCC. Our work had uncovered that macrophages play a crucial role in linking NASH and HCC. In addition, we had created an SPCG model and identified MED8, YBX1, and KPNA2 as specific prognostic markers for HCC.

Recently, there has been a growing emphasis on investigating the tumor immune microenvironment in various research [54, 55]. This heightened attention is due to the potential therapeutic benefits that can be derived from targeting this specific milieu. As a result, these approaches provide a broader selection of clinical therapy options. The hepatic tumor microenvironment comprises a diverse array of cellular components, including macrophages, HSCs, hepatocytes, T cells, B cells, neutrophils, and fibroblasts [56]. Our research involving enrichment analysis of NASH revealed a significant increase in damage-promoting pathways, particularly in macrophages. We detected the activation of multiple proinflammatory and anti-injury pathways in our investigation. It should be noted that HSCs showed activation in NASH, and there was a significant increase in the EMT pathway (Additional file 2: Fig. S1A), which plays a crucial role in liver injury and fibrosis [57]. The JAK/STAT3 signaling pathway is essential for regulating cell proliferation and differentiation. The activation of this pathway has been demonstrated to have a role in the formation of a tumor inflammatory environment [58]. The results of our investigation indicated a notable upregulation in the IL-6-JAK-STAT3 signaling pathway in macrophages and neutrophils in the HCC group.

HSCs, functioning as intrahepatic cells, release cytokines that contribute to the progression of HCC by facilitating communication between immune cells [59]. HSCs boost the growth of Extracellular matrix (ECM) by producing tissue inhibitor of metal protease 1 (TIMP-1), leading to the creation of a tumor matrix that facilitates tumor cells in evading the immune system [59]. Consistent with earlier studies [60], our research findings (Additional file 2: Fig. S4B, G) confirmed that HSCs have a greater abundance in NASH. Furthermore, the consistent recognition of HSCs as a vital component in NASH has been discovered using several techniques [61, 62]. The occurrence of HCC is frequently concomitant with hepatic fibrosis, indicating a significant involvement of liver fibrosis in the development of HCC. HSCs are commonly acknowledged as the main source of cancer-associated fibroblasts (CAFs) [63]. The activation of HSCs in NASH triggers the synthesis of ECM by fibroblasts, ultimately leading to the development of liver fibrosis [64]. A recent study [65] demonstrated that CAFs can inhibit hepatocyte apoptosis by increasing the Bcl-2/BAX ratio through the SDF1/CXCR2/PI3K/AKT signaling pathway. The relationship between the FOXQ1/N-Myc downstream gene 1 (NDRG1) axis and the occurrence of HCC, as well as the existence of CAFs, has been demonstrated [66]. CAFs also have a role in promoting angiogenesis and EMT by releasing cytokines [67]. This discovery offers a rationale for the documented rise in HSCs in the NASH dataset during our study (Additional file 1: Fig. S4B, G).

The Scissor analysis revealed the presence of malignant hepatocytes, T cells, and macrophages as positive cells. T cells, when continuously exposed to the malignant environment, demonstrate a decrease in their original immunological and tumor regulatory capabilities [68]. This leads to a condition known as T cell exhaustion, which is linked to decreased patient survival rates [69]. Furthermore, the prognostic significance of bidirectional activation resulting from the interaction between T cells and B cells has been extensively seen in HCC patients [70]. When examining the pathogenesis of NASH and HCC, the abundance of macrophages was found to be significantly increased in several combination analyses. It may be shown that macrophages have a more prominent involvement in comparison to T cells. TAMs have been identified as key contributors to the advancement of HCC by aiding its spread and encouraging EMT [71], related mechanisms include hypoxia-inducible factor 1-alpha (HIF-1α)/IL-1β/TLR4 [72] and Janus kinase 2 (JAK2)/STAT3/Snail [73] pathways. This aligns with our finding that the occurrence of EMT coincides with the spatial distribution of macrophages in the same area (refer to Figs. 4 and 5).

This study demonstrated that the MIF signaling pathway functions as the primary ligand-receptor pair that facilitates the interactions between hepatocytes and macrophages (as depicted in Figs. 3 and 5). This discovery confirmed the essential significance of the MIF signaling pathway in the progression of both NASH and HCC. The immunomodulatory cytokine MIF has a notable influence on both innate and adaptive immunity [74, 75]. MIF can also decrease fibrogenic HSC activation through the CD47/AMP-activated protein kinase (AMPK) signaling pathway [76]. Additionally, it exhibits a hepatoprotective action by partially suppressing steatosis [77, 78]. The potential of MIF as a biomarker has been recognized, as its elevated levels have been linked to unfavorable outcomes in various types of cancer [79–81]. Furthermore, it has been suggested that MIF has the potential to serve as a diagnostic sign for colorectal cancer [82]. Within the context of HCC, MIF assumes a tumor-promoting role by enhancing proliferation and inhibiting apoptosis, a mechanism likely facilitated by the interaction between MIF and ERK1/2 [75]. The role of the MIF signaling pathway varies in the development of NASH and HCC, highlighting the importance of accurately identifying it before NASH progresses to HCC.

In an attempt to establish a more precise prognostic model for HCC, we integrated a gene set derived from the Scissor algorithm. This approach was employed to overcome the limitations associated with single-cell models. Following a step-by-step screening process that involved univariate Cox, LASSO-Cox, and multivariate Cox regression analysis, three genes were selected for the development of the SPCG model. The accuracy of the model was evaluated by utilizing ICGC-LIRI-JP clinical cohorts, and the findings obtained from the difference tests were deemed satisfactory. Notably, the SPCG model established in this study showed exceptional predictive capabilities for patients diagnosed with either NASH or HCC. The mediator complex subunit 8 (MED8) has been shown to play a crucial role in transcription as a regulator of polymerase activity. The level of MED8 expression was observed to be elevated in HCC tissues. The suppression of MED8 led to a notable decrease in the proliferation and migration of HepG2 and Huh7 cells [83]. Further investigation has elucidated that MED8 plays a crucial role in determining a poor prognosis in HCC, principally by promoting cancer progression through the activation of TH2 cytokines [84]. YBX1, sometimes referred to as YB-1, is a versatile RNA-binding protein that possesses the evolutionarily conserved Cold-shock Domain (CSD) [85]. YBX1 participates in diverse biological processes and possesses the capacity to modulate a broad spectrum of genes that govern cell proliferation, cell viability, resistance to drugs, and instability of chromatin [86]. Karyopherin α2 (KPNA2), a protein implicated in the conventional pathway of nuclear protein transportation, has been observed in many instances of cancer, including HCC [87, 88], Nevertheless, the exact molecular mechanisms responsible for the function of KPNA2 are still not fully understood. Previous studies have shown that KPNA2 promotes the advancement of HCC by interfering with the cell cycle and increasing the expression of CCNB2 (cyclin B2)/CDK1 (cyclin-dependent kinase 1). On the other hand, inhibiting KPNA2 could potentially halt the progression of the cell cycle [89]. Recent research has revealed that the overexpression of Long non-coding RNA (LncRNA) HAGLROS selectively targets the miR-26b-5p/KPNA2 signaling pathway, leading to the inactivation of p53. This molecular mechanism ultimately contributes to the progression of HCC [90]. Furthermore, the signaling pathway consisting of KDM4A-AS1/KPNA2/HIF-1α plays a significant role in the proliferation and metastasis of HCC [91]. The capacity to merge diverse information, aided by developments in algorithms for assessing single-cell and spatial transcriptomes, allows for the development of models that function at the level of individual cells. This technique offers substantial insights that are extremely important for therapeutic intervention, early identification of NASH, and proactive detection of HCC occurrences.

## Conclusion

To summarize, our study clarified the connection between NASH and HCC using a thorough analysis method that combined single-cell, bulk, and spatial transcriptomics. An SPCG model was created to specifically identify MED8, YBX1, and KPNA2 as individual prognostic markers for HCC. The SPCG model has demonstrated considerable promise in forecasting outcomes for patients with HCC, and its efficacy has been bolstered by rigorous validation. Furthermore, our research has revealed that macrophages are important disease-causing factors in NASH and HCC at the single-cell level. By making this discovery, we have been able to create a prognostic model for liver cancer and improve the precision of identifying NASH. The incorporation of various omics techniques has yielded a comprehensive comprehension of the cellular interactions and environment implicated in the progression of NASH and HCC. These findings have important consequences for the development of enhanced diagnostic and prognostic techniques.

### Supplementary Information

**Additional file 1: Table S1.** Primers used in this study. **Table S2.** Expression programs detected by cNMF in each of the 32 samples, Related to Fig. 3. **Table S3.** Meta-programs, each derived from multiple related cNMF programs, ordered from most to least significant, Related to Fig. 4. **Table S4.** Gene intersection between multiple groups, Related to Fig. 7. **Table S5.** Results of univariate Cox regression screening, Related to Fig. 7. **Table S6.** Results of Lasso-COX regression screening, Related to Fig. 7. **Table S7.** Results of multivariate Cox regression screening, Related to Fig. 7.

**Additional file 2: Fig. S1.** HALMAKER pathway enrichment. HALMAKER pathway enrichment in NASH (up, GSE129516) and HCC (down, GSE142868). **Fig. S2.** Heatmap showing the contribution of signals. **A-B** Heatmap of incoming (A) and outgoing (B) signaling pathways in NASH dataset (GSE129516). The upper colored bar graph represents the cumulative signaling intensity of a cell group by totaling all signaling pathways represented in the heatmap. The right-hand grey bar graph indicates the overall signaling strength of a signaling pathway by adding up all cell groups exhibited in the heatmap. **C-D** Heatmap of incoming (C) and outgoing (D) signal reception pathways in HCC (GSE142868). The upper colored bar graph represents the cumulative signaling intensity of a cell group by totaling all signaling pathways represented in the heatmap. The right-hand grey bar graph indicates the overall signaling strength of a signaling pathway by adding up all cell groups exhibited in the heatmap. **Fig. S3.** Annotating macrophage subclusters and performing cytoTRANCE analysis. **A-B** Detecting specific markers for subgroups of macrophages. The dot size indicates the fraction of expressing cells, and the dots are colored based on average expression levels. NASH (GSE129516, A), HCC (GSE142868, B).**C-D** CytoTRACE predicts the ordering of macrophage subgroups based on their developmental potential, from the lowest differentiation ability to the highest. NASH (GSE129516, C), HCC (GSE142868, D). **Fig. S4.** Immune cell infiltration and GSEA enrichment analysis combined with bulk RNA-seq dataset. **A, C** Wilcoxon test of the immune cell infiltration differential analysis based on the ssGSEA algorithm in the NASH dataset (GSE129516, A) and HCC dataset (GSE142868, C). Significance is denoted as follows: ns indicates nonsignificance; * p < 0.05; ** p < 0.01; *** p < 0.001; **** p < 0.0001. **B, D** Stacking plot depicting the proportion of immune cells based on the CIBERSORTX algorithm in the NASH dataset (GSE129516, B) and HCC dataset (GSE142868, D). **E.** Volcano map displaying differential gene expression using bulk RNA-seq dataset. **F–H** Gene set enrichment analysis (GSEA) based on single-cell gene sets and RNA-seq in the NASH dataset (GSE129516, F, G) and HCC dataset (GSE142868, H), NES > 0, activated; NES < 0, inhibited. **Fig. S5.** Dot plot showing the cluster-specific marker genes.** A** Cell marker detection of HCC single-cell dataset. The dot size indicates the fraction of expressing cells, and the dots are colored based on average expression levels**. B** Detection of cell markers for macrophage subsets. The dot size indicates the fraction of expressing cells, and dots are colored based on average expression levels. **Fig. S6.** Application of CellChat to calculated signal communication in HCC single cell dataset (GSE151530). **A** Heatmap shows the relative importance of each cell group based on the computed four network centrality measures of the MIF signaling network.** B** All the significant ligand-receptor pairs that contribute to the signal transmission from hepatocytes to other cell types. Dots represent the contribution of each receptor pair in signals emitted by hepatocytes toward various cells in HCC patients. Dot size indicates significance, while color shade represents the magnitude of contribution. Darker shades, particularly red, indicate a higher contribution, while lighter shades indicate a lower contribution**. C-D** Heatmap of outgoing (C) and incoming (D) signaling pathways in the HCC (Human) dataset. The upper colored bar graph represents the cumulative signaling intensity of a cell group by totaling all signaling pathways represented in the heatmap. The right-hand grey bar graph indicates the overall signaling strength of a signaling pathway by adding up all cell groups exhibited in the heatmap. **Fig. S7.** Uivariate and multivariate COX regression analysis, as well as clinical correlation and survival analysis of three independent prognostic genes in HCC patients. **A-B** Univariate (A) and multivariate (B) COX regression analysis of the signature and different clinical features. **C-F** Pathological stage (C), Sex (D), pathological grade (E), and Age (F), survival analysis of patients in the relevant high-low risk group **G** Survival curves related to YBX1, KPNA2, and MED8 expression levels in the high- and low-risk groups.

## Data Availability

All data used in this study are publicly available as described in the Materials and Methods section. Bulk transcriptomic data (GSE199105) and single-cell transcriptomic data (GSE129516) for mouse nonalcoholic fatty liver disease were obtained from the GEO database (https://www.ncbi.nlm.nih.gov/geo/). Bulk transcriptomic data (GSE50431) and single-cell transcriptomic data (GSE142868) for mouse HCC were retrieved from the GEO database. Human single-cell transcriptomic data were obtained from the GEO database (GSE151530). The training and validation datasets for clinical model construction were from the TCGA database (https://portal.gdc.cancer.gov/) and the ICGC database (https://dcc.icgc.org/), respectively. Four spatial transcriptomic datasets are available in the Zenodo database (https://zenodo.org/). The study cohort for NASH was GSE89632. Other further inquiries can be directed to the corresponding author.

## Abbreviations

ANOVA: One-way analysis of variance
AUC: Area under the curve
BP: Biological processes
CAFs: Cancer-associated fibroblasts
cDC: Conventional Dendritic cell
cNMF: Consensus Non-negative Matrix factorization
DC: Dendritic cell
DEGs: Differentially expressed genes
ECM: Extracellular matrix
EIP: Environmental Information Processing
EMT: Epithelial-mesenchymal transition
Endo: Endothelial cell
ER: Endoplasmic reticulum
Fib: Fibroblast
GEO: Gene Expression Omnibus
GMT: Gene Matrix Transposed file format
GO: Gene Ontology
GSEA: Gene-set enrichment analysis
HCC: Hepatocellular carcinoma
HD: Human Disease
Hep: Hepatocyte
HSC: Hepatic Stellate Cell
ICGC: International Cancer Genome Consortium
KEGG: Kyoto Encyclopedia of Genes and Genomes
K-M: Kaplan‒Meier
Macro: Macrophage
MIF: Macrophage migration inhibitory factor
MsigDB: Molecular Signatures Database
NAFLD: Nonalcoholic fatty liver disease
NASH: Nonalcoholic steatohepatitis
NES: Normalized enrichment score
Neu: Neutrophil
NK: Natural killer cell
NK T: Natural killer T cell
OS: Organismal Systems
Plasma: Plasma cell
ROC: Receiver operating characteristic
ROS: Reactive oxygen species
SPCG: Scissor Positive cell Correlated Genes
ssGSEA: Single sample gene set enrichment analysis
TAM: Tumor-associated macrophage
TCGA: The Cancer Genome Atlas
TMB: Tumor mutation burden
Tregs: Regulatory T cells
t-SNE: T-distributed Stochastic Neighbor Embedding
UMAP: Uniform Manifold Approximation and Projection
