# Supplementary figures and images for: Unraveling the underlying pathogenic factors driving nonalcoholic steatohepatitis and hepatocellular carcinoma: an in-depth analysis of prognostically relevant gene signatures in hepatocellular carcinoma
Source: J Transl Med. 2024 Jan 18;22:72. doi: 10.1186/s12967-024-04885-6 (PMC10795264; doi:10.1186/s12967-024-04885-6)

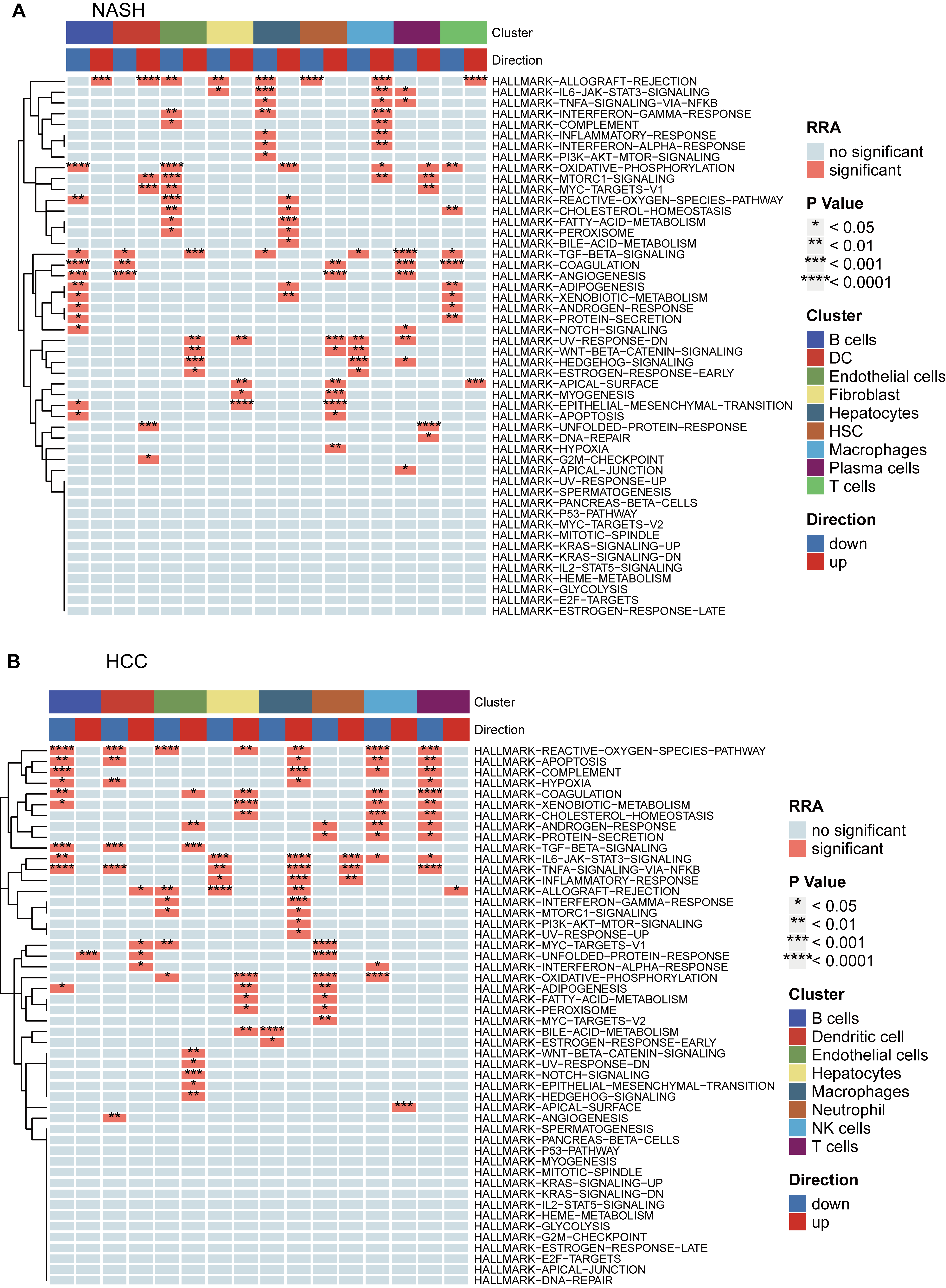

Supplement: Supplementary file 2 — Additional file 2: Fig. S1. HALMAKER pathway enrichment. HALMAKER pathway enrichment in NASH (up, GSE129516) and HCC (down, GSE142868). Fig. S2. Heatmap showing the contribution of signals. A-B Heatmap of incoming (A) and outgoing (B) signaling pathways in NASH dataset (GSE129516). The upper colored bar graph represents the cumulative signaling intensity of a cell group by totaling all signaling pathways represented in the heatmap. The right-hand grey bar graph indicates the overall signaling strength of a signaling pathway by adding up all cell groups exhibited in the heatmap. C-D Heatmap of incoming (C) and outgoing (D) signal reception pathways in HCC (GSE142868). The upper colored bar graph represents the cumulative signaling intensity of a cell group by totaling all signaling pathways represented in the heatmap. The right-hand grey bar graph indicates the overall signaling strength of a signaling pathway by adding up all cell groups exhibited in the heatmap. Fig. S3. Annotating macrophage subclusters and performing cytoTRANCE analysis. A-B Detecting specific markers for subgroups of macrophages. The dot size indicates the fraction of expressing cells, and the dots are colored based on average expression levels. NASH (GSE129516, A), HCC (GSE142868, B).C-D CytoTRACE predicts the ordering of macrophage subgroups based on their developmental potential, from the lowest differentiation ability to the highest. NASH (GSE129516, C), HCC (GSE142868, D). Fig. S4. Immune cell infiltration and GSEA enrichment analysis combined with bulk RNA-seq dataset. A, C Wilcoxon test of the immune cell infiltration differential analysis based on the ssGSEA algorithm in the NASH dataset (GSE129516, A) and HCC dataset (GSE142868, C). Significance is denoted as follows: ns indicates nonsignificance; * p < 0.05; ** p < 0.01; *** p < 0.001; **** p < 0.0001. B, D Stacking plot depicting the proportion of immune cells based on the CIBERSORTX algorithm in the NASH dataset (GSE129516, B) and HC [file 12967_2024_4885_MOESM2_ESM.zip › Fig S1.tif]

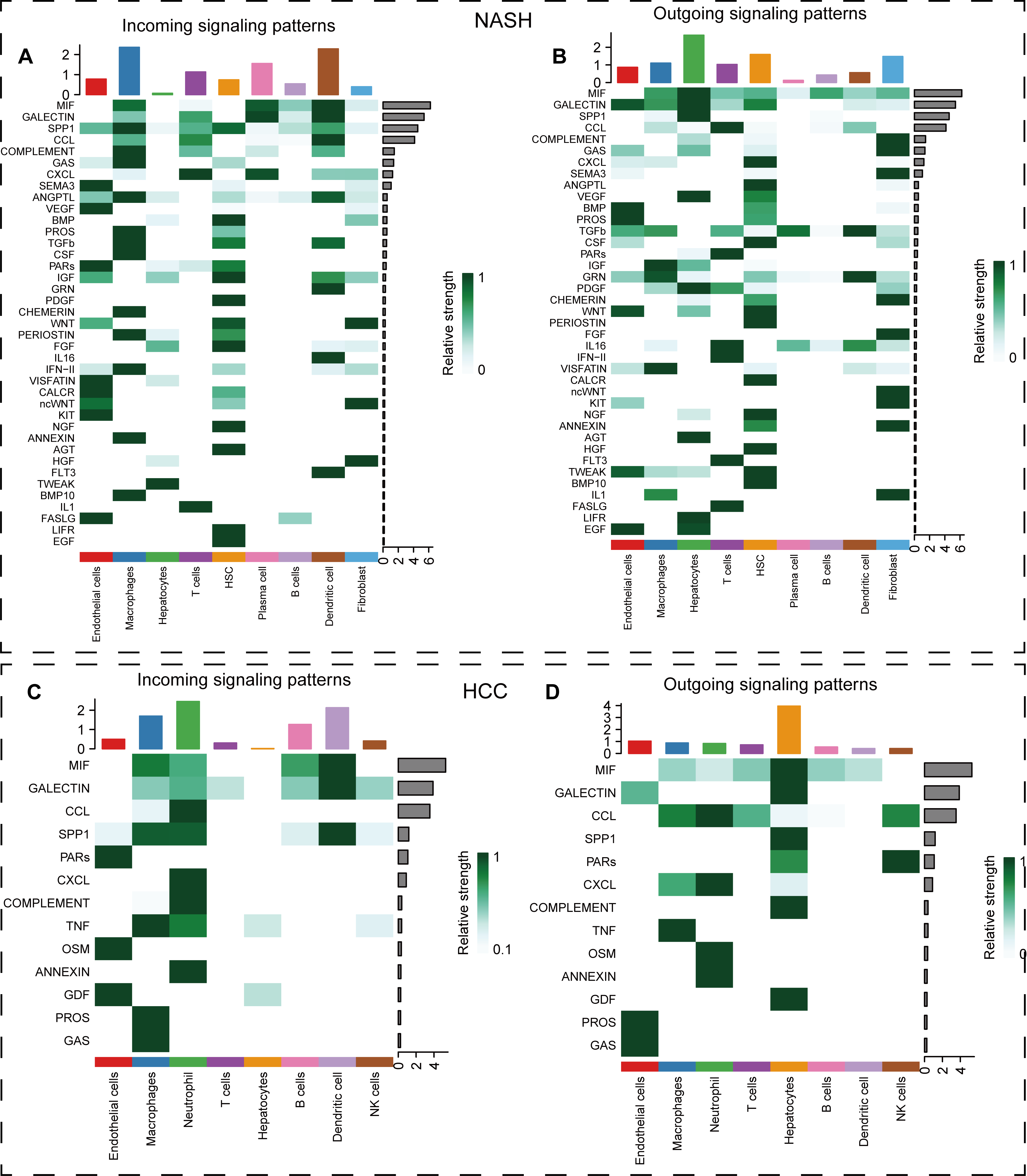

Supplement: Supplementary file 2 — Additional file 2: Fig. S1. HALMAKER pathway enrichment. HALMAKER pathway enrichment in NASH (up, GSE129516) and HCC (down, GSE142868). Fig. S2. Heatmap showing the contribution of signals. A-B Heatmap of incoming (A) and outgoing (B) signaling pathways in NASH dataset (GSE129516). The upper colored bar graph represents the cumulative signaling intensity of a cell group by totaling all signaling pathways represented in the heatmap. The right-hand grey bar graph indicates the overall signaling strength of a signaling pathway by adding up all cell groups exhibited in the heatmap. C-D Heatmap of incoming (C) and outgoing (D) signal reception pathways in HCC (GSE142868). The upper colored bar graph represents the cumulative signaling intensity of a cell group by totaling all signaling pathways represented in the heatmap. The right-hand grey bar graph indicates the overall signaling strength of a signaling pathway by adding up all cell groups exhibited in the heatmap. Fig. S3. Annotating macrophage subclusters and performing cytoTRANCE analysis. A-B Detecting specific markers for subgroups of macrophages. The dot size indicates the fraction of expressing cells, and the dots are colored based on average expression levels. NASH (GSE129516, A), HCC (GSE142868, B).C-D CytoTRACE predicts the ordering of macrophage subgroups based on their developmental potential, from the lowest differentiation ability to the highest. NASH (GSE129516, C), HCC (GSE142868, D). Fig. S4. Immune cell infiltration and GSEA enrichment analysis combined with bulk RNA-seq dataset. A, C Wilcoxon test of the immune cell infiltration differential analysis based on the ssGSEA algorithm in the NASH dataset (GSE129516, A) and HCC dataset (GSE142868, C). Significance is denoted as follows: ns indicates nonsignificance; * p < 0.05; ** p < 0.01; *** p < 0.001; **** p < 0.0001. B, D Stacking plot depicting the proportion of immune cells based on the CIBERSORTX algorithm in the NASH dataset (GSE129516, B) and HC [file 12967_2024_4885_MOESM2_ESM.zip › Fig S2.tif]

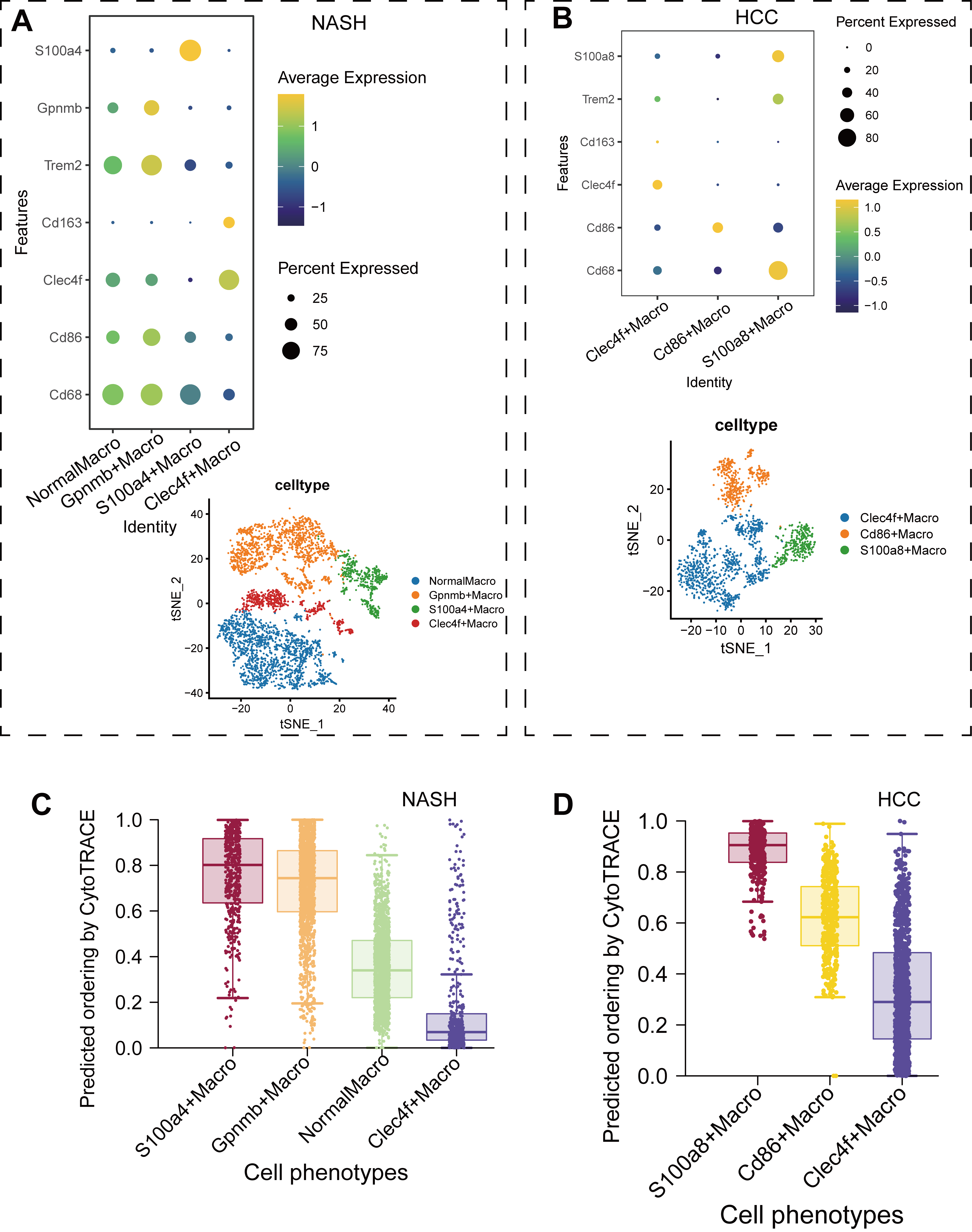

Supplement: Supplementary file 2 — Additional file 2: Fig. S1. HALMAKER pathway enrichment. HALMAKER pathway enrichment in NASH (up, GSE129516) and HCC (down, GSE142868). Fig. S2. Heatmap showing the contribution of signals. A-B Heatmap of incoming (A) and outgoing (B) signaling pathways in NASH dataset (GSE129516). The upper colored bar graph represents the cumulative signaling intensity of a cell group by totaling all signaling pathways represented in the heatmap. The right-hand grey bar graph indicates the overall signaling strength of a signaling pathway by adding up all cell groups exhibited in the heatmap. C-D Heatmap of incoming (C) and outgoing (D) signal reception pathways in HCC (GSE142868). The upper colored bar graph represents the cumulative signaling intensity of a cell group by totaling all signaling pathways represented in the heatmap. The right-hand grey bar graph indicates the overall signaling strength of a signaling pathway by adding up all cell groups exhibited in the heatmap. Fig. S3. Annotating macrophage subclusters and performing cytoTRANCE analysis. A-B Detecting specific markers for subgroups of macrophages. The dot size indicates the fraction of expressing cells, and the dots are colored based on average expression levels. NASH (GSE129516, A), HCC (GSE142868, B).C-D CytoTRACE predicts the ordering of macrophage subgroups based on their developmental potential, from the lowest differentiation ability to the highest. NASH (GSE129516, C), HCC (GSE142868, D). Fig. S4. Immune cell infiltration and GSEA enrichment analysis combined with bulk RNA-seq dataset. A, C Wilcoxon test of the immune cell infiltration differential analysis based on the ssGSEA algorithm in the NASH dataset (GSE129516, A) and HCC dataset (GSE142868, C). Significance is denoted as follows: ns indicates nonsignificance; * p < 0.05; ** p < 0.01; *** p < 0.001; **** p < 0.0001. B, D Stacking plot depicting the proportion of immune cells based on the CIBERSORTX algorithm in the NASH dataset (GSE129516, B) and HC [file 12967_2024_4885_MOESM2_ESM.zip › Fig S3.tif]

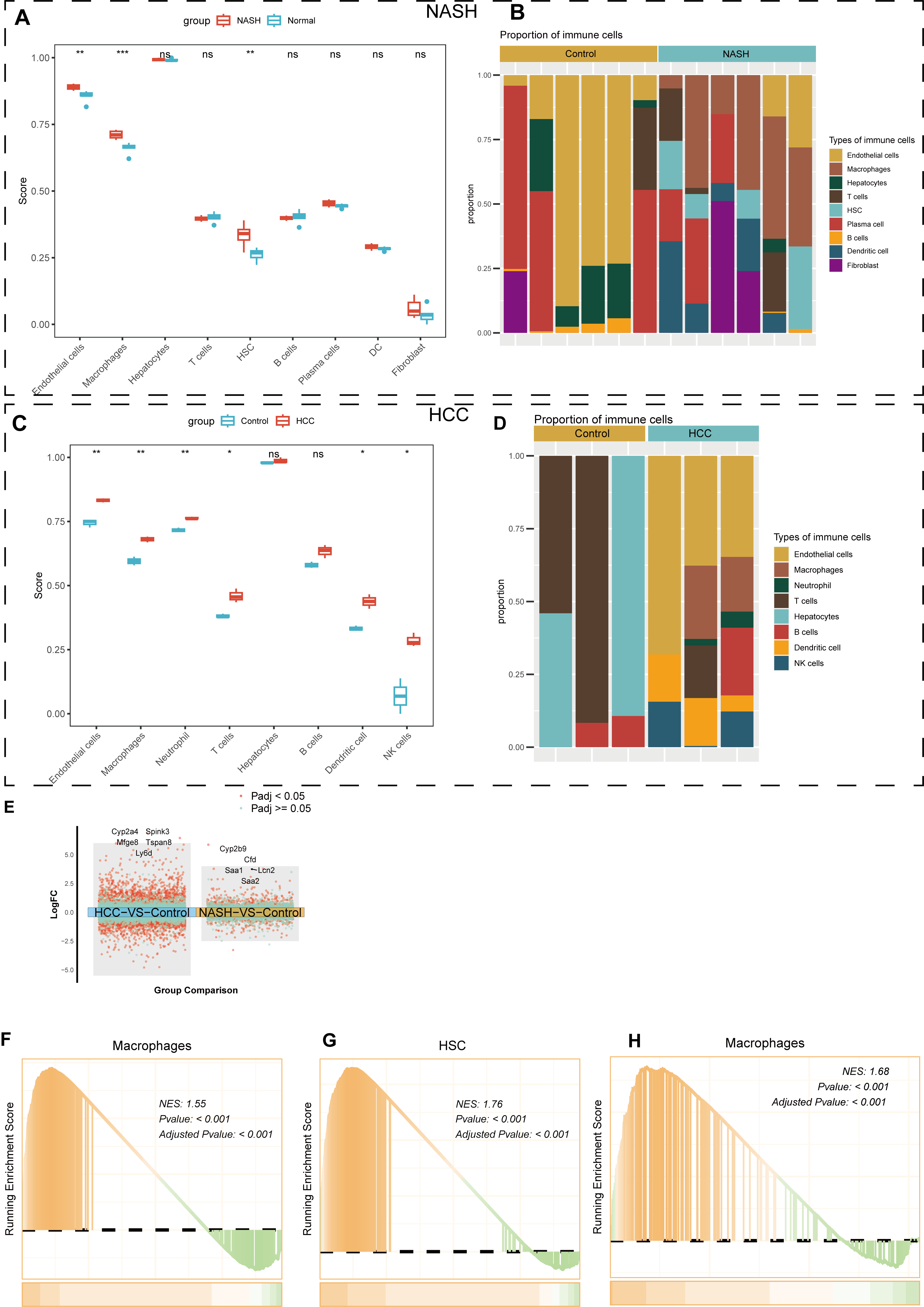

Supplement: Supplementary file 2 — Additional file 2: Fig. S1. HALMAKER pathway enrichment. HALMAKER pathway enrichment in NASH (up, GSE129516) and HCC (down, GSE142868). Fig. S2. Heatmap showing the contribution of signals. A-B Heatmap of incoming (A) and outgoing (B) signaling pathways in NASH dataset (GSE129516). The upper colored bar graph represents the cumulative signaling intensity of a cell group by totaling all signaling pathways represented in the heatmap. The right-hand grey bar graph indicates the overall signaling strength of a signaling pathway by adding up all cell groups exhibited in the heatmap. C-D Heatmap of incoming (C) and outgoing (D) signal reception pathways in HCC (GSE142868). The upper colored bar graph represents the cumulative signaling intensity of a cell group by totaling all signaling pathways represented in the heatmap. The right-hand grey bar graph indicates the overall signaling strength of a signaling pathway by adding up all cell groups exhibited in the heatmap. Fig. S3. Annotating macrophage subclusters and performing cytoTRANCE analysis. A-B Detecting specific markers for subgroups of macrophages. The dot size indicates the fraction of expressing cells, and the dots are colored based on average expression levels. NASH (GSE129516, A), HCC (GSE142868, B).C-D CytoTRACE predicts the ordering of macrophage subgroups based on their developmental potential, from the lowest differentiation ability to the highest. NASH (GSE129516, C), HCC (GSE142868, D). Fig. S4. Immune cell infiltration and GSEA enrichment analysis combined with bulk RNA-seq dataset. A, C Wilcoxon test of the immune cell infiltration differential analysis based on the ssGSEA algorithm in the NASH dataset (GSE129516, A) and HCC dataset (GSE142868, C). Significance is denoted as follows: ns indicates nonsignificance; * p < 0.05; ** p < 0.01; *** p < 0.001; **** p < 0.0001. B, D Stacking plot depicting the proportion of immune cells based on the CIBERSORTX algorithm in the NASH dataset (GSE129516, B) and HC [file 12967_2024_4885_MOESM2_ESM.zip › Fig S4.tif]

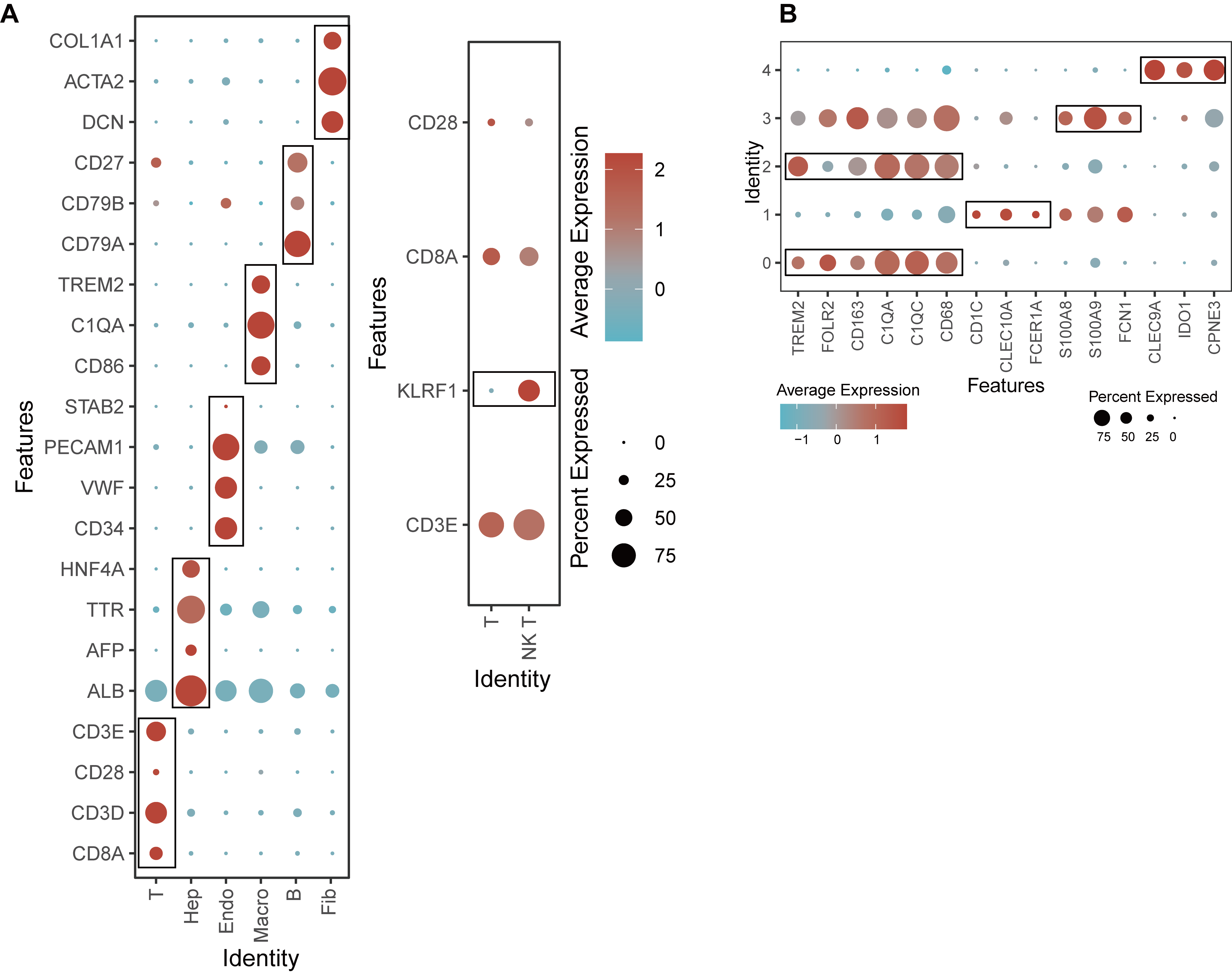

Supplement: Supplementary file 2 — Additional file 2: Fig. S1. HALMAKER pathway enrichment. HALMAKER pathway enrichment in NASH (up, GSE129516) and HCC (down, GSE142868). Fig. S2. Heatmap showing the contribution of signals. A-B Heatmap of incoming (A) and outgoing (B) signaling pathways in NASH dataset (GSE129516). The upper colored bar graph represents the cumulative signaling intensity of a cell group by totaling all signaling pathways represented in the heatmap. The right-hand grey bar graph indicates the overall signaling strength of a signaling pathway by adding up all cell groups exhibited in the heatmap. C-D Heatmap of incoming (C) and outgoing (D) signal reception pathways in HCC (GSE142868). The upper colored bar graph represents the cumulative signaling intensity of a cell group by totaling all signaling pathways represented in the heatmap. The right-hand grey bar graph indicates the overall signaling strength of a signaling pathway by adding up all cell groups exhibited in the heatmap. Fig. S3. Annotating macrophage subclusters and performing cytoTRANCE analysis. A-B Detecting specific markers for subgroups of macrophages. The dot size indicates the fraction of expressing cells, and the dots are colored based on average expression levels. NASH (GSE129516, A), HCC (GSE142868, B).C-D CytoTRACE predicts the ordering of macrophage subgroups based on their developmental potential, from the lowest differentiation ability to the highest. NASH (GSE129516, C), HCC (GSE142868, D). Fig. S4. Immune cell infiltration and GSEA enrichment analysis combined with bulk RNA-seq dataset. A, C Wilcoxon test of the immune cell infiltration differential analysis based on the ssGSEA algorithm in the NASH dataset (GSE129516, A) and HCC dataset (GSE142868, C). Significance is denoted as follows: ns indicates nonsignificance; * p < 0.05; ** p < 0.01; *** p < 0.001; **** p < 0.0001. B, D Stacking plot depicting the proportion of immune cells based on the CIBERSORTX algorithm in the NASH dataset (GSE129516, B) and HC [file 12967_2024_4885_MOESM2_ESM.zip › Fig S5.tif]

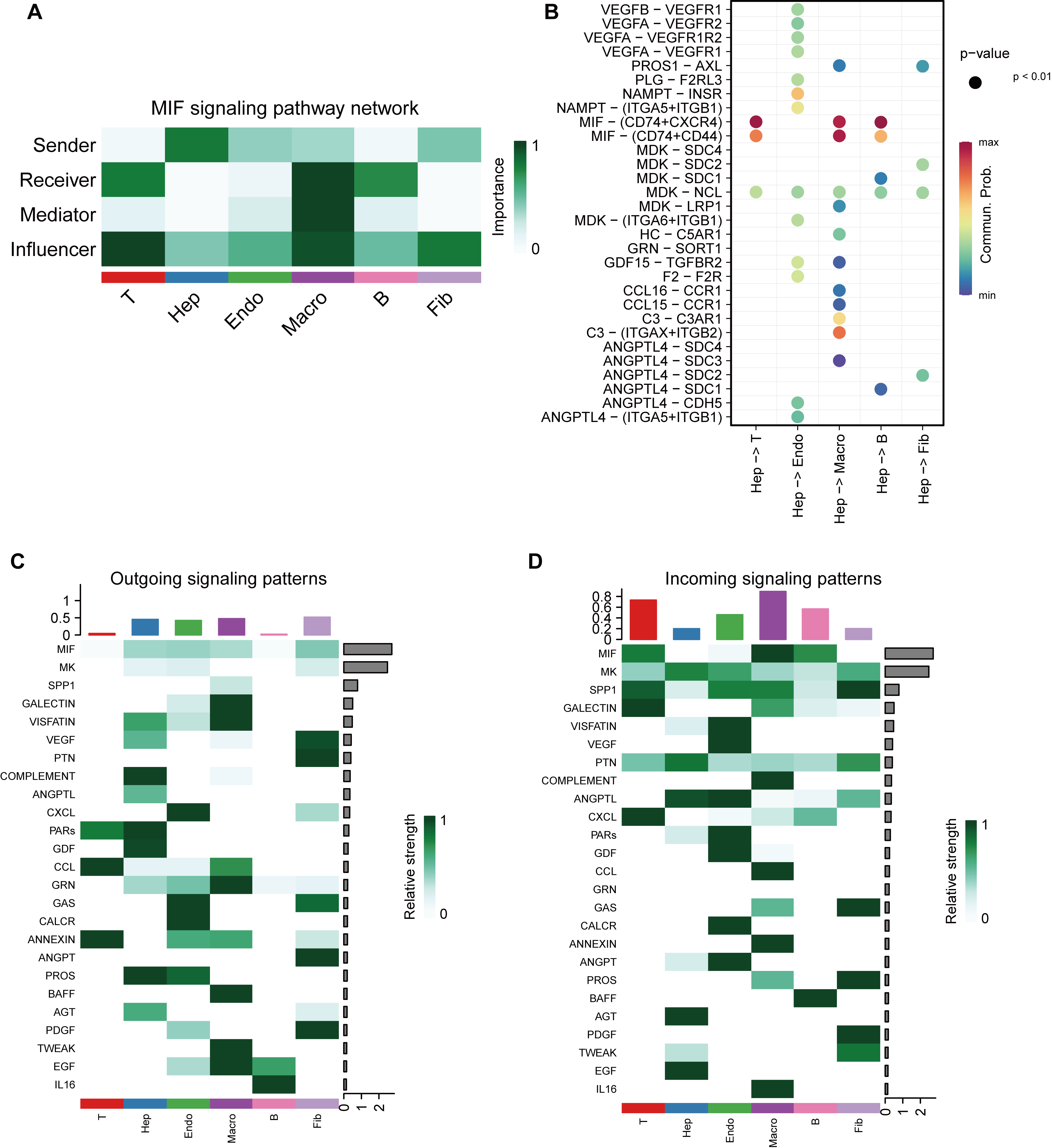

Supplement: Supplementary file 2 — Additional file 2: Fig. S1. HALMAKER pathway enrichment. HALMAKER pathway enrichment in NASH (up, GSE129516) and HCC (down, GSE142868). Fig. S2. Heatmap showing the contribution of signals. A-B Heatmap of incoming (A) and outgoing (B) signaling pathways in NASH dataset (GSE129516). The upper colored bar graph represents the cumulative signaling intensity of a cell group by totaling all signaling pathways represented in the heatmap. The right-hand grey bar graph indicates the overall signaling strength of a signaling pathway by adding up all cell groups exhibited in the heatmap. C-D Heatmap of incoming (C) and outgoing (D) signal reception pathways in HCC (GSE142868). The upper colored bar graph represents the cumulative signaling intensity of a cell group by totaling all signaling pathways represented in the heatmap. The right-hand grey bar graph indicates the overall signaling strength of a signaling pathway by adding up all cell groups exhibited in the heatmap. Fig. S3. Annotating macrophage subclusters and performing cytoTRANCE analysis. A-B Detecting specific markers for subgroups of macrophages. The dot size indicates the fraction of expressing cells, and the dots are colored based on average expression levels. NASH (GSE129516, A), HCC (GSE142868, B).C-D CytoTRACE predicts the ordering of macrophage subgroups based on their developmental potential, from the lowest differentiation ability to the highest. NASH (GSE129516, C), HCC (GSE142868, D). Fig. S4. Immune cell infiltration and GSEA enrichment analysis combined with bulk RNA-seq dataset. A, C Wilcoxon test of the immune cell infiltration differential analysis based on the ssGSEA algorithm in the NASH dataset (GSE129516, A) and HCC dataset (GSE142868, C). Significance is denoted as follows: ns indicates nonsignificance; * p < 0.05; ** p < 0.01; *** p < 0.001; **** p < 0.0001. B, D Stacking plot depicting the proportion of immune cells based on the CIBERSORTX algorithm in the NASH dataset (GSE129516, B) and HC [file 12967_2024_4885_MOESM2_ESM.zip › Fig S6.tif]

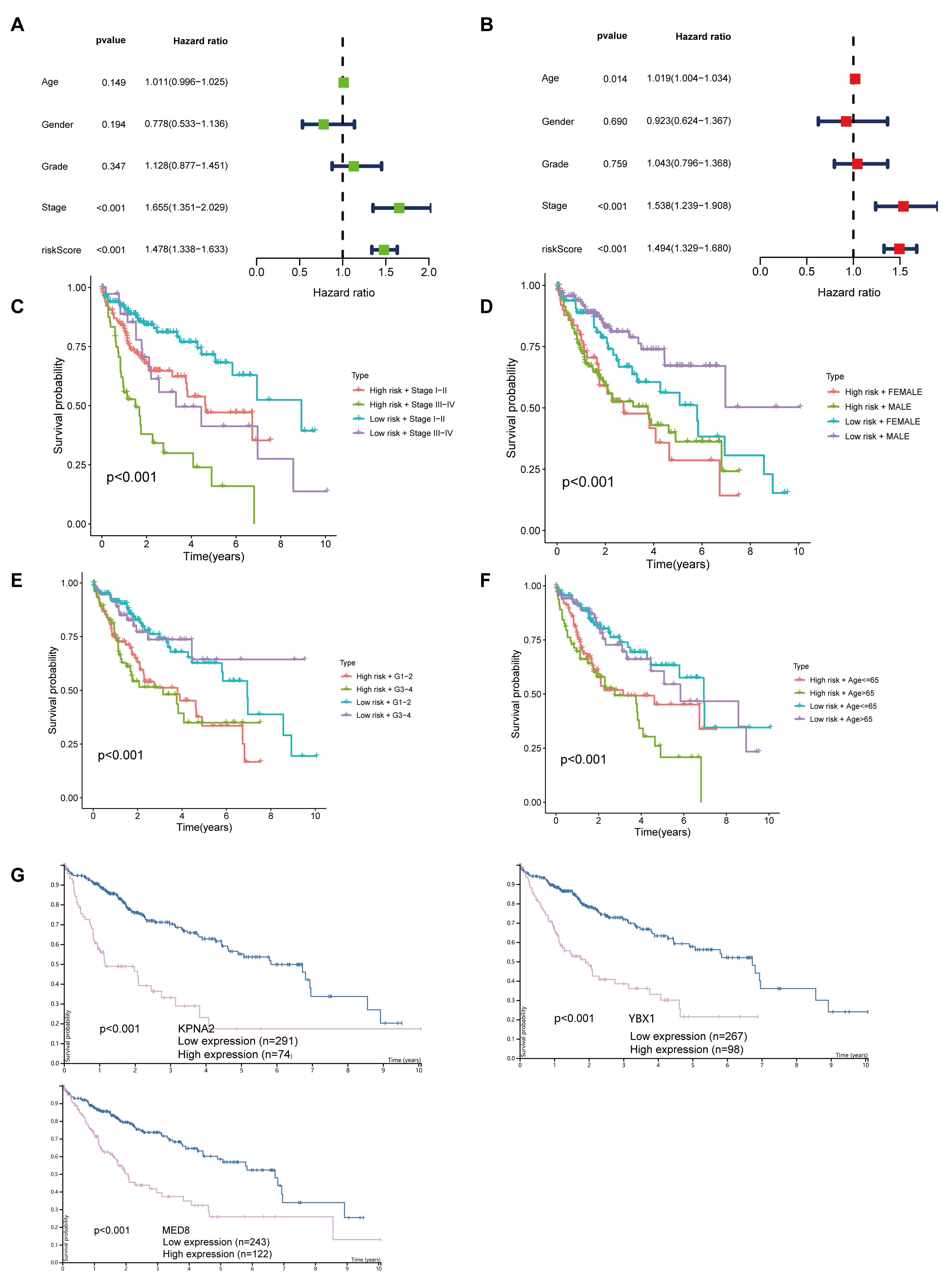

Supplement: Supplementary file 2 — Additional file 2: Fig. S1. HALMAKER pathway enrichment. HALMAKER pathway enrichment in NASH (up, GSE129516) and HCC (down, GSE142868). Fig. S2. Heatmap showing the contribution of signals. A-B Heatmap of incoming (A) and outgoing (B) signaling pathways in NASH dataset (GSE129516). The upper colored bar graph represents the cumulative signaling intensity of a cell group by totaling all signaling pathways represented in the heatmap. The right-hand grey bar graph indicates the overall signaling strength of a signaling pathway by adding up all cell groups exhibited in the heatmap. C-D Heatmap of incoming (C) and outgoing (D) signal reception pathways in HCC (GSE142868). The upper colored bar graph represents the cumulative signaling intensity of a cell group by totaling all signaling pathways represented in the heatmap. The right-hand grey bar graph indicates the overall signaling strength of a signaling pathway by adding up all cell groups exhibited in the heatmap. Fig. S3. Annotating macrophage subclusters and performing cytoTRANCE analysis. A-B Detecting specific markers for subgroups of macrophages. The dot size indicates the fraction of expressing cells, and the dots are colored based on average expression levels. NASH (GSE129516, A), HCC (GSE142868, B).C-D CytoTRACE predicts the ordering of macrophage subgroups based on their developmental potential, from the lowest differentiation ability to the highest. NASH (GSE129516, C), HCC (GSE142868, D). Fig. S4. Immune cell infiltration and GSEA enrichment analysis combined with bulk RNA-seq dataset. A, C Wilcoxon test of the immune cell infiltration differential analysis based on the ssGSEA algorithm in the NASH dataset (GSE129516, A) and HCC dataset (GSE142868, C). Significance is denoted as follows: ns indicates nonsignificance; * p < 0.05; ** p < 0.01; *** p < 0.001; **** p < 0.0001. B, D Stacking plot depicting the proportion of immune cells based on the CIBERSORTX algorithm in the NASH dataset (GSE129516, B) and HC [file 12967_2024_4885_MOESM2_ESM.zip › Fig S7.tif]
